# Advances in fully integrated wearable near-infrared spectroscopy: hardware innovations and biomedical applications

**DOI:** 10.1088/2515-7647/ae6ae4

**Published:** 2026-05-28

**Authors:** Shashikant Lahade, Nicholas Ross, Thomas D O’Sullivan

**Affiliations:** 1 Department of Electrical Engineering, University of Notre Dame, Notre Dame, IN 46556, United States of America; 2 Bioengineering Program, University of Notre Dame, Notre Dame, IN 46556, United States of America

**Keywords:** near-infrared spectroscopy, NIRS, fNIRS, wearables, continuous health monitoring

## Abstract

The use of noninvasive near-infrared spectroscopy (NIRS) has grown significantly over the past few decades for use in the characterization of tissue optical properties and derived physiological parameters such as tissue oxygen saturation and hemoglobin composition to support human health and wellness. Wearable NIRS devices have been developed in recent years that enable real-time continuous monitoring. These devices have been enabled by advances in the dense integration of miniature integrated circuits, light sources and detectors, embedded processing, and wireless technologies. The most attractive aspect of wearable NIRS devices is that they enable real-time sensing both inside and outside of laboratory and clinical settings. This review paper focuses on recent hardware developments related to fully integrated wearable NIRS devices, including continuous wave, time domain, and frequency domain NIRS techniques, and their enabling technologies. We also review their application in biomedical fields such as neuroscience, musculoskeletal physiology, and others. We provide our perspective on the future technology research opportunities and direction of the field.

## 1 Introduction

Near-infrared spectroscopy (NIRS) is a non-invasive sensing and imaging technique that is used to investigate deep tissue (up to several centimeters) molecular composition and function using safe levels of visible and near-infrared light. Compared to other noninvasive biomedical imaging modalities, NIRS benefits include the use of non-ionizing radiation, relatively low-cost instrumentation, and high sensitivity to tissue optical absorbers such as hemoglobin, water, fat, and collagen without the need for administration of a contrast agent. NIRS commonly takes advantage of the strong red to near-infrared optical absorption of hemoglobin to characterize hemodynamics (oxy- and deoxy-hemoglobin concentrations) and tissue oxygen saturation, which is the ratio of oxyhemoglobin (HbO) to total hemoglobin concentration in bulk tissue (i.e. including both arterial and venous components). It is thus valuable for assessing tissue viability, perfusion, and oxygen metabolism. Studying hemodynamic activity, for example, provides insights into brain function and neural activity, enabling the assessment of cognitive and neural health in children and adults.

Jöbsis first demonstrated the ability to measure tissue deoxyhemoglobin (HHb) and HbO using NIRS in 1977 [1], utilizing the ‘first biological window’ in the range of 650–1000 nm. This regime avoids significant absorption from hemoglobin at shorter wavelengths as well as water at longer wavelengths, allowing for photon paths that can exceed 100 mm. However, because the scattering coefficient in tissue is typically much higher than the absorption coefficient (about 10x or more), multiple scattering results in a depth sensitivity of up to a few centimeters [2]. Several groups have also explored the use of longer shortwave infrared (SWIR) light up to 2000 nm to take advantage of less scattering in this range and enhanced sensitivity to water, lipid, and collagen [3–5].

NIRS devices have been used in clinical studies since the inception of the technique, beginning with systems that were large and required a dedicated laboratory bench or cart. Their large size, complexity, and limited mobility restricted their usage in long-term continuous monitoring, especially for applications in naturalistic environments outside of clinical and laboratory settings including home based and consumer applications. For example, they were not suitable for deployment as point-of-care tools for first responders, in physician offices, or at athletic facilities, as their large size would necessitate a dedicated exam room in a clinic or imaging facility. Likewise, tethered NIRS devices required users to remain stationary during measurements, restricting their mobility and the practicality of long-term continuous monitoring while decreasing comfort. This, combined with their complexity, has also stifled their deployment in limited resource environments that could benefit from such cost-effective technologies.

In contrast, wearable NIRS devices can offer several advantages that address these limitations and have the potential to dramatically expand the research, healthcare, and consumer applications of NIRS. Wearable devices can cater to the growing need for continuous and non-invasive monitoring in health, sports, and neuroscience. The advantages of wearable NIRS devices include real-time, continuous, and long-term data acquisition as well as improved patient comfort. Real-time data collection and analysis allows for immediate insight and intervention, promotes proactive healthcare management, and aids in the development of personalized treatment plans. Patients and physicians would benefit from continuous monitoring that provides a more complete picture of health status for diagnosis and treatment rather than static or intermittent snapshots. Remote monitoring is also a promising application of wearable devices, especially in light of the COVID-19 pandemic, where minimizing physical contact was crucial.

In addition to the potential health and research benefits of wearable devices, they have a tremendous economic impact. In 2020, wearable technology held a global market size of ∼$44.3 billion. It more than doubled to $98.5 billion in 2025 and is estimated to grow to $186 billion by 2030, with smartwatches and fitness trackers driving the growth (most of which have some sort of optical sensing)[6]. Based on this and the potential for improved clinical outcomes, it is evident that additional research is warranted into the development and application of more capable wearable devices utilizing NIRS.

### 1.1 Purpose and scope

**Figure 1.**
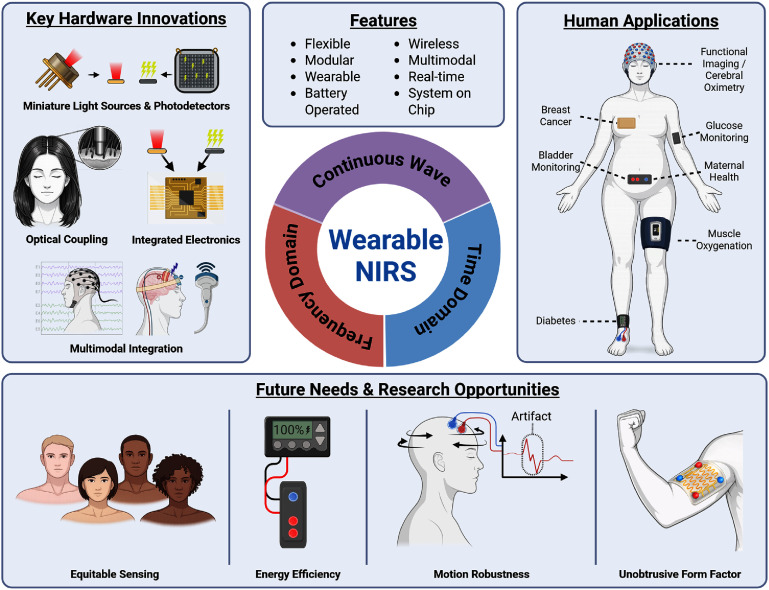
Landscape of wearable near-infrared spectroscopy (NIRS) technology research, applications, and opportunities.

The goal of this topical state-of-the-art review paper is to provide a comprehensive, multidisciplinary synthesis of the current progress in wearable NIRS technology, as graphically illustrated in figure 1. Specifically, we evaluate the evolution of device architectures and their underlying enabling components, noting that while most commercially available wireless systems currently utilize continuous wave (CW)-NIRS for its low complexity and cost-effectiveness, recent advancements have successfully enabled wearable quantitative frequency domain (FD)-NIRS and time domain (TD)-NIRS systems. Beyond hardware, we highlight the diverse range of applications enabled by these advancements—from uses in exercise monitoring to ambulatory neuroimaging—and provide a critical perspective on remaining technical challenges and future research opportunities. By integrating these disparate areas of development, this review aims to serve as a roadmap for device engineers, NIRS researchers, and clinicians in the field.

To compose this review, we identified relevant papers using multiple literature databases and discovery tools including Google Scholar, Web of Science, PubMed, and Litmaps [7]. Searches were conducted across the NIRS hardware, signal processing, and biomedical application domains, as well as through forward and backward citation tracing of identified works. We generally limited the scope of our review to (1) papers published in the past 20 years (i.e. since 2006) and (2) wearable, untethered and wireless NIRS devices that enable unconstrained movement. Exceptions to these criteria were made for foundational studies predating 2006 that provide necessary historical context, as well as tethered systems that have significantly advanced component-level miniaturization directly relevant to modern wearable NIRS development.

Given the vast work in wearable optical sensing, to balance comprehensiveness and length, our review does not cover closely related NIRS modalities including devices that solely perform photoplethysmography (PPG) and coherence-based flow sensing such as diffuse correlation spectroscopy (DCS) and speckle contrast optical spectroscopy. We refer the interested reader to recent reports on these modalities (PPG: [8–11] and DCS: [12]). Also, although NIRS is a model-based sensing modality that is inextricably linked to the theory of light-tissue interactions, we also primarily focus on hardware developments rather than theoretical and computational advances. There are several excellent reviews on NIRS as a whole that discuss the theory of light-tissue interactions and the various models that are used to model them [13–16].

### 1.2 Outline

This review paper is organized as follows. We begin by covering the working principles for each of the three main NIRS techniques (CW, TD, and FD). Subsequently, we discuss the design considerations for wearable NIRS, including hardware architectures and components, as well as approaches for optimizing wearability and function. Then, we review recent demonstrations of CW-, TD-, and FD-NIRS wearable devices, including commercialized and multi-modality devices. We explore promising human applications of wearable NIRS, before presenting our future outlook for wearable NIRS technologies, including potential challenges and research opportunities.

## 2 Types of NIRS technologies: CW-, FD-, & TD-NIRS

NIRS is commonly categorized into three types based upon the temporal characteristics of the tissue-interrogating light source: CW-NIRS, FD-NIRS, and TD-NIRS. Despite their different implementations, all three methods share a common measurement paradigm: near-infrared light (typically 650–1000 nm) is delivered into tissue, undergoes multiple scattering and absorption events, and emerging photons are collected by photodetectors positioned at one or more source-detector separations (SDSs) in a reflectance or transmission geometry. The fundamental difference between these techniques lies in how they encode and extract information about tissue optical properties from the detected light. In the following subsections, we discuss the basis and practical characteristics of each approach, highlighting their capabilities and limitations in the context of wearable device implementation. In addition, table 1 summarizes the key characteristics of each of these NIRS implementations.

**Table 1.** Comparison of CW-, FD-, and TD-NIRS approaches across key system characteristics.

Characteristic	CW-NIRS	TD-NIRS	FD-NIRS
Measures absolute optical properties ($\mu_a, \mu_s^{^{\prime}}$)	Possible, but difficult using spatially resolved techniques	Yes	Yes

Detector types	Si PIN photodiodes, APDs, SiPMs	SPADs, SiPMs, PMTs^a^	APDs, SiPMs, PMTs^a^

Sources	LEDs, laser diodes, & VCSELs	Laser diodes & VCSELs	Laser diodes & VCSELs

Source drivers	DC to (slow) hundreds of kHz modulation for ambient light rejection	High-speed pulse generators ($ {\gt} $400 ps pulse width); 20–100 MHz repetition rate	High-speed RF modulation circuits (50–1000 MHz)

Data acquisition	Low-noise 16/24-bit ADCs; sampling rates in the Hz to kHz range	TCSPC or time-gated FPGA	Demodulation and phase-sensitive detection

System complexity	Low; minimal components required, enabling compact and low-cost designs	High; TCSPC chain (CFD, TAC, ADC), pulsed laser drivers, and timing electronics increase complexity	Moderate–High; requires precise RF synthesis, phase detection, and demodulation electronics

Wearable maturity	High	Medium	Low

^a^ Common in benchtop systems but unsuitable for wearables due to large size and high bias required. *Terms: ADC—analog to digital converter; APD—avalanche photodiode; CFD—constant fraction discriminator; DC—direct current; FPGA—field programmable gate array; LED—light emitting diode; PMT—photomultiplier tube; PWM—pulse width modulation; RF—radio frequency; Si PIN—silicon p-intrinsic-n type photodiode; SiPM—silicon photomultiplier; SPAD—single-photon avalanche diode; TAC—time to amplitude converter; TCSPC—time-correlated single-photon counting; VCSEL—vertical-cavity surface-emitting laser*

### 2.1 CW

**Figure 2.**
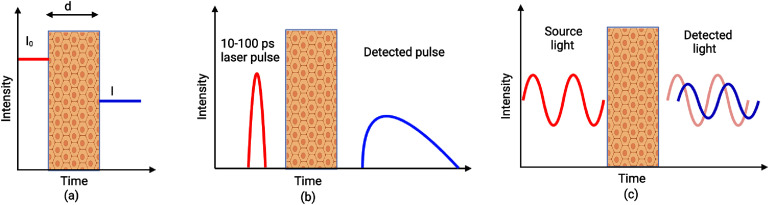
NIRS measurement domains: (a) continuous wave (CW), (b) time domain (TD), and (c) frequency domain (FD) [17]. Created in BioRender. O’Sullivan, T. (2026) https://BioRender.com/g37j098.

As illustrated in figure 2(a), CW-NIRS employs constant or near-constant intensity light to interrogate tissue. Light sources can be modulated (typically up to hundreds of kHz) to enable lock-in detection that improves signal-to-noise ratio (SNR) and rejects ambient light. It is important to note that this modulation frequency range is practically too low to extract temporal information about photon propagation [18]. As a result, CW is most often used to monitor changes in tissue optical absorber (i.e. chromophore) concentrations assuming fixed optical scattering, which is accomplished using the modified Beer–Lambert law [2]. Due to its relatively low cost, and simple hardware requirements, CW-NIRS is the most widely adopted technique in wearable NIRS devices. However, the inherent difficulty in using CW-NIRS to separate absorption from scattering and to provide absolute quantification represents significant constraints for applications that require longitudinal comparisons between sessions or individuals where variations in scattering are expected to be significant.

### 2.2 TD

TD-NIRS employs short laser pulses ($ {\gt} $400 ps) to interrogate tissue. Using fast, single-photon counting photodetectors, TD-NIRS works by measuring the photon distribution time-of-flight (DTOF), commonly referred to as the temporal point spread function (TPSF), in tissue at sub-nanosecond resolution (figure 2(b)) [14]. Analyzing the dispersion of these short pulses through tissue allows one to separate the effects of and quantify both optical absorption and scattering [2, 19]. Time gating can also be used in TD-NIRS to isolate and interrogate deeper in tissue with less dependence upon SDS than other NIRS approaches, and it has been shown to be less sensitive to skin tone [20]–a notable contribution given the longstanding concerns regarding skin tone bias in optical imaging which we discuss further in section 6.1.2. Because of its dependence on highly-sensitive detectors, TD-NIRS systems are inherently sensitive to ambient light leakage, and TD-NIRS has historically come at the expense of additional complexity and cost. However, recent advances have begun to make TD-NIRS devices more accessible, as we will describe in section 4.2 [14].

### 2.3 FD

Rather than using laser pulses to interrogate tissue, FD-NIRS uses intensity-modulated laser light, typically in the range of 50–1000 MHz, in what can be thought of (somewhat simplistically) as the Fourier equivalent of TD-NIRS [2, 18]. Like TD-NIRS, FD-NIRS can also quantify tissue optical properties by characterizing both absorption and scattering simultaneously. The intensity-modulated source light in FD-NIRS induces photon density waves (PDWs) in multiple-scattering tissue, which are composed of individual photons. As these individual photons encounter absorption and scattering events, they cause an attenuation of the amplitude and delay in phase of the overall PDW relative to the source/origin, as shown in figure 2(c). Absorption and scattering can be quantitatively estimated by regressing the detected PDW amplitude and phase (strictly speaking, the complex-valued reflectance) relative the source and, after instrument response calibration, to a FD model of light-tissue interactions (e.g. diffusion approximation to the radiative transport equation). Several groups have shown that the phase component offers deeper tissue sensitivity than CW-NIRS [21, 22]. However, the requirement for RF modulation, phase-sensitive detection, and precise frequency control increases the complexity and size of the system. As discussed in section 4.3, these factors have historically posed challenges for the implementation of wearable FD-NIRS, although, like TD-NIRS, recent advances are beginning to address these limitations.

## 3 Design considerations for wearable NIRS device

Fully wearable NIRS devices require the miniature integration of light sources, detectors, and electronics for sensing, as well as a battery and data storage or a wireless transceiver. In this section, we discuss these key components, highlighting technical considerations specific to each NIRS technique (CW-, TD-, or FD-), as well as approaches to assure good tissue optical coupling. We then review approaches to improving device wearability, including user comfort and device durability, before examining the effects and correction of motion artifacts (MAs). By exploring these aspects, we aim to provide a thorough understanding of the technological constraints and challenges involved in creating effective and practical wearable NIRS devices.

### 3.1 Hardware

#### 3.1.1 Optical sources and driver circuits

The choice of light source type in NIRS systems is driven by application-specific requirements such as wavelength, optical power, modulation bandwidth, power efficiency, and device footprint. Semiconductor light emitting diodes (LEDs) and laser diodes are both well-suited for wearable form factors due to their small size, low cost, and power efficiency, but both require careful thermal management because rising temperatures can reduce optical output and shift emission wavelength. LEDs are available in a wide range of wavelengths from the visible to SWIR with sufficient optical power for NIRS, making them the typical source for CW-NIRS. Dual-wavelength LED packages, such as the Epitex L750/850-04A (Japan) and the Marubeni SMT735D/850D (USA), are deployed in CW-NIRS systems as a means of reducing overall footprint. Due to their tiny size, microLEDs are also advantageous for skin-conformable NIRS devices [23]. However, LEDs exhibit broader emission bandwidths (typically tens of nanometers) than laser diodes ($ {\lt} 1$ nm), which can increase effective wavelength uncertainty and spectral crosstalk during chromophore unmixing if the system spectral response is not calibrated or corrected [24]. Most LEDs are not suitable for FD-NIRS as their modulation bandwidth is typically limited to much less than the minimum ∼50 MHz [25] required to induce a measurable modulation phase shift. Similar constraints apply to TD-NIRS and although sub-nanosecond pulsed LED modules have recently become available, they are bulky and ill-suited for wearable applications at this time.

Laser diodes offer narrow spectral and high modulation bandwidths, making them broadly compatible with wearable FD- and TD-NIRS when safety and thermal considerations are appropriately managed. Both traditional edge-emitting laser diodes, such as the Thorlabs L690P010 (USA), and vertical-cavity surface-emitting lasers (VCSELs), such as the ams-OSRAM OLI2020V.A1-850-A (Austria), can provide sufficient optical power up to and exceeding the ANSI Z136.1 maximum permissible exposure for skin. Like microLEDs, VCSELs are particularly attractive for wearables due to their small die size and simpler packaging, but their commercial availability is limited to a handful of relevant wavelengths. A unique aspect of VCSELs is that higher powers can be achieved with multiple apertures and multiple wavelengths can be co-packaged together to create bright compact multi-wavelength light sources, which is advantageous for higher SNR and increasing spectral content [26–28].

Pulsed laser diodes, one example being the PicoQuant LDH series (Germany), are suitable for TD-NIRS since they offer pulse widths in the range of 50–100 ps with repetition rates (i.e. optical pulses emitted per second) between 20–100 MHz; however, custom pulsed laser diodes are typically sought out as commercial products are too bulky for wearable implementations [29–31]. VCSELs can also be used for picosecond pulse generation at high repetition rates for TCSPC [32, 33]. Recent implementations have demonstrated fully integrated probe-level designs with VCSELs directly bonded to application-specific integrated circuit (ASIC) timing electronics [28, 32], which we will discuss in more detail in section 4.2.1. However, some challenges to pulsed laser diodes can include long warm up times to achieve picosecond range pulse timing stability (∼60 min), their temperature sensitivity causing variations affecting pulse characteristics (e.g. width, timing precision), and typically larger driver electronics than their CW counterparts [14].

In NIRS devices, LED/laser drivers regulate the source current to maintain stable optical output despite power variations such as battery droop. This is commonly done with miniature integrated circuit (IC)-based drivers. ICs can control optical power by maintaining a set drive current (current control), or by adjusting the drive current to maintain a fixed optical power (power control) using a reference detector. Source drivers should provide sufficient current output for the light source (upwards of ∼100 mA for laser diodes) as well as stable and low noise operation (microamp level). Additional desirable features include soft start and brownout protection to prevent current transients that could damage the light source, allow for easily adjustable current settings that can be configured via external components (e.g. analog or digital control), and have multiple channels to control the desired number of sources. For example, the MLD203CLN (Thorlabs, USA) is a low-cost, low-noise, high-precision laser diode driver, and the TLC5940 (Texas Instruments, USA) is a compact multichannel LED driver used in wearable NIRS devices.

#### 3.1.2 Photodetectors

High-sensitivity photodetectors are critical for measuring the highly-attenuated light that results after propagation through tissue. The sensitivity of NIRS devices is primarily limited by its detector performance. Larger SDSs can result in deeper tissue sensitivity, but at the expense of lower signal. Therefore a highly sensitive detector is desirable to maximize depth sensitivity. The responsivity of a photodetector (output current per incident optical power) must be balanced against the required temporal response, as the two characteristics are inherently in conflict. Large area detectors detect more photons but exhibit more capacitance which results in a lower frequency response. Thus the selection of suitable detector technologies varies depending upon the desired NIRS technique: CW, TD, or FD. Beyond bandwidth and responsivity, thermal stability is crucial in wearable applications because temperature variations due to ambient changes or direct skin contact can directly affect noise characteristics, gain, and photon detection efficiency (PDE) [34]. In particular, photodetector dark current increases with temperature [35], degrading SNR. Fluctuations in temperature can also cause dramatic variations in the breakdown voltage of silicon photomultipliers (SiPMs) and avalanche photodiodes (APDs), leading to lower PDE and inconsistent gain that can reduce measurement stability. Table 2 summarizes these characteristics—gain, bandwidth, bias requirements, and thermal sensitivity—across the four detector technologies most commonly deployed in wearable NIRS systems.

**Table 2.** Comparison of photodetectors relevant to wearable NIRS systems. Wearable suitability ratings reflect the aggregate impact of voltage requirements, power consumption, and form factor.

Detector type	Internal gain	Bandwidth / timing resolution	Bias voltage	Dynamic range	Noise sources & other nonidealities	Wearable suitability
PIN Photodiode	Unity (1×)	High; $ {\gt} $1000 MHz possible; larger detector area reduces bandwidth	Low: 5–30 V reverse bias	Very high ($ {\gt} $100 dB); highly linear; rarely saturates under typical NIRS conditions	Shot noise and thermal noise dominant; dark currents ($ {\gt} $1 nA typical); low NEP	Very high

APD (Avalanche photodiode)	50–100× (linear mode)	High; $ {\gt} $1000 MHz possible; larger detector area reduces bandwidth	High: 100–400 V (Si APD)	Moderate (∼50–80 dB); limited by gain-dependent excess noise and avalanche saturation	Excess noise from avalanche multiplication process; amplified dark current; gain and breakdown voltage are temperature-sensitive; requires thermal stabilization	Low

SPAD (Single-photon avalanche diode)	${\sim} 10^{5}$–10^6^ (Geiger mode; binary pulse output)	Timing jitter 10–100 ps (IRF); count rate limited by dead time (1–100 ns per event)	Moderate: 20–70 V	Limited by dead time; max ${\sim} 10^{7}$–10^8^ cps; prone to saturation under ambient light; smaller SDS needed for high-count-rate operation	Dark count rate (DCR); afterpulsing probability; optical crosstalk (in arrays); no analog excess noise (binary output); DCR increases exponentially with temperature	High

SiPM (Silicon photomultiplier)	${\sim} 10^{5}$–10^6^ (parallel SPAD array; analog sum output)	Several kHz to tens of MHz; limited by microcell RC time constant; lower than PIN or APD at equal detector area	Moderate: 40–75 V	Moderate; determined by microcell count (100–10k cells); non-linear at high photon flux	Summed DCR across all microcells; inter-cell optical crosstalk; afterpulsing; gain and breakdown voltage are temperature-sensitive; bias temperature compensation may be necessary in wearable devices	High

Miniaturized spectrometer	Unity (CMOS photodiode array; no internal gain)	Sub-Hz to kHz depending on pixel count and integration time	Low: 3.3–5 V	Moderate (∼80 dB) for single integration time; effectively larger by adjusting integration time	Read noise; circuit noise; dark output (increases with integration time and ambient temperature); stray light from grating imperfections and surface reflections	Low–Moderate

*Terms: APD—avalanche photodiode; CMOS—complementary metal-oxide-semiconductor; cps—counts per second; DCR—dark count rate; IRF—instrument response function; NEP—noise equivalent power; NIRS—near-infrared spectroscopy; PIN—p-intrinsic-n photodiode; SDS—source-detector separation; SiPM—silicon photomultiplier; SPAD—single-photon avalanche diode.*

Silicon (Si) PIN photodiodes, such as the Vishay Intertechnology VEMD5160X01 (USA), are widely used in CW-NIRS devices due to their large-area (e.g. ${\unicode{x2A7E}} 1$ mm^2^), low dark current, high linear dynamic range, and wide spectral response up to ∼1100 nm [36, 37]. The co-packaged or monolithic integration of a photodiode with a transimpedance amplifier (TIA), such as the Texas Instruments OPT101 (USA), has been used to reduce component footprint, and can improve noise performance by minimizing current leakage, noise pick-up, and stray capacitance [38–41]. However, the low bandwidth of large area photodiodes and their inability to detect single photons make them unsuitable for FD- and TD-NIRS implementations.

APDs attracted interest for use in CW- and FD-NIRS as an intermediate detector class between linear PIN photodiodes and single-photon devices like photomultiplier tubes (PMTs) [13, 42]. When operated below breakdown in linear avalanche mode, APDs provide internal gain via impact ionization (typically 50–100×) while maintaining an analog output proportional to incident optical power [42, 43]. This gain means higher sensitivity compared to PIN photodiodes without requiring photon counting, making APDs well suited for FD-NIRS systems that depend on accurate recovery of modulation amplitude and phase. Compact APD packages, notably Hamamatsu Photonics S2384 and S17268-05 (Japan), and CMOS-integrated APD front ends with co-designed TIAs have further supported miniaturization [44]. However, their high bias voltage requirements (100–400 V) require bulky voltage converters, and complicate isolation and safety in skin-contact devices, limiting adoption in fully wearable NIRS platforms.

Single-photon APDs (SPADs) (e.g. the Hamamatsu Photonics C16534-050GD (Japan) and the Singular Photonics Andarta sensor (Scotland)) operate in Geiger mode, where the device is biased above breakdown such that a single photon can trigger an avalanche of charge carriers that produce a measurable current pulse [45]. Consequently, optical information is encoded in photon arrival timing and count statistics rather than a continuous analog signal. This operating mode makes SPADs particularly well suited for TD-NIRS, where precise photon time-of-flight measurements enable depth discrimination via time gating and suppression of early, superficially scattered photons [14]. When combined with fast-gating electronics and on-chip or external time-to-digital converters (TDCs), SPAD-based systems achieve excellent temporal resolution, low noise-equivalent power (NEP), and compact form factors suitable for wearable TD instrumentation [28, 32, 33]. However, SPADs face intrinsic and system-level limitations, including dead-time-limited dynamic range, elevated dark count and afterpulsing noise, and increased readout complexity and cost when scaling to large arrays [33, 45].

Recently, SiPM detectors, which consists of hundreds to thousands of SPADs monolithically connected in parallel, have been integrated into CW-, TD-, and FD-NIRS devices [14, 30, 43, 46–49]. Due to their modest bias voltage requirements (40–75 V) and miniature footprint, devices such as the Hamamatsu Photonics S13360 series and S15639 (Japan) are desirable intrinsically amplified photodetector compatible with a wearable form factor [32, 43]. Because of their high sensitivity, SiPMs can provide substantial SNR improvements compared to single APDs in photon-limited regimes; one FD-NIRS study using the S13360 series reported an increase of 30 dB [43]. However, SiPMs comparatively have a smaller dynamic range and bandwidth than APDs and photodiodes. Their limited dynamic range results in saturation at high optical powers, which must be monitored and compensated for by, for example, reducing the source power or increasing the SDS. The lower bandwidth of SiPMs can limit response at the high modulation frequencies required for FD-NIRS, however, it is compensated by the SiPM’s higher gain (∼10^6^).

Photodetectors that are sensitive to longer SWIR wavelengths and compatible with wearable NIRS include indium gallium arsenide (InGaAs)-based PIN photodiodes, APDs, and SPADs [50]. InGaAs photodiodes, such as the Edmund Optics 17-076 (USA), have large bandwidths with cutoff frequencies exceeding 10 GHz and have been successfully deployed in wearable multi-wavelength SWIR probes [4]. Similarly to their silicon counterparts, InGaAs APDs, for example the Excelitas Technologies C30662 (USA), offer higher sensitivity than PIN photodiodes, though their photosensitive areas are limited to a few hundred micrometers, which reduces sensitivity to weak signals. A critical limitation of InGaAs detectors is their high levels of thermally induced dark current that inherently arise from their low energy bandgap [50]. Elevated dark current degrades SNR and raises the NEP, limiting achievable SDS and depth sensitivity without cooling. Thermoelectric coolers, while effective, can consume several watts of power, which is a significant fraction of a wearable device’s total power budget, to achieve the required cooling (−10 ^∘^C to −20 ^∘^C) [50]. Such power requirements would drastically reduce battery life or require the use of a larger battery, thus limiting its wearability.

Broadband spectrometers have been used as a way to increase spectral content in NIRS devices [51]. Using diffractive elements or hyperspectral imaging arrays, spectrometers measure the light intensity as a function of wavelength over a specific designed range. Traditionally, these devices have been bulky and unsuitable for wearable platforms. Recent efforts, however, have resulted in the availability of miniature, coin-sized spectrophotometers that can be easily integrated into wearable NIRS devices, as demonstrated with the Hamamatsu Photonics C14384MA (Japan) in [51]. The increase in spectral content can be used to characterize additional tissue components, such as cytochrome-c-oxidase activity.

For completeness, many benchtop TD- and FD-NIRS systems utilize PMTs as fiber-coupled detectors due to their high gain and single photon counting capabilities (for TD), but they are bulky, require a high bias ($ {\gt} 1000$ V), and have a limited dynamic range that requires frequent gain adjustment. They are therefore not suitable for low profile wearable NIRS devices.

#### 3.1.3 Processing, communications, and power

NIRS devices rely on a controller and supporting devices to manage the measurement and data collection, with options including microcontrollers (e.g. Espressif Systems ESP32 (China) [36] and Microchip Technology AtMega16A (USA) [52]), digital signal processors, single-board computers (e.g. Raspberry Pi (United Kingdom)) [53], and field-programmable gate arrays (FPGAs) (e.g. Advanced Micro Devices Artix 7 (USA)) for high performance, high channel count, or timing-critical designs [42, 54, 55]. Arduino-based platforms are also used for rapid prototyping and education-oriented development [39].

Interfaces between controllers and peripherals typically use serial protocols such as SPI or I2C. SPI generally supports higher throughput but requires more chip-select and data lines, whereas I2C reduces wiring by sharing a two-wire bus across peripherals at the cost of lower speed and potential bus contention. As optode count increases (e.g. to fully cover the human head), microcontrollers often require general-purpose input/output expanders [56], switches, or multiplexers to control optode sequencing. For high-density systems, FPGAs such as the Advanced Micro Devices Artix-7 and Zynq-7000 (USA) are particularly well-suited because programmable logic can implement custom rapid sequencing, demodulation, and parallel data paths that increase sampling rates ($\unicode{x2A7E}$100 Hz) and provide real-time on-board data processing [42, 57, 58].

The choice of analog-to-digital conversion (ADC) hardware and operation is critical to ensuring accurate and precise measurements, while balancing power consumption and heat generation. High-resolution ADCs ensure that small changes in light absorption can be accurately captured, which is crucial for precisely measuring variations in the pulsatile waveform [36, 46]. The sampling rate of the ADC determines the frequency with which the analog signal is measured and converted into digital form. The sampling rate should be chosen to capture the targeted dynamics while avoiding unnecessary power consumption, heat generation, and data transfer bottlenecks. Low-power, high-resolution delta-sigma (ΔΣ) ADCs, such as the Texas Instruments ADS1298 and ADS1299 (USA), are frequently used for wearable devices due to their ability to extend battery life while retaining data quality [59–61].

Wearable NIRS devices generally use Bluetooth or Wi-Fi communication standards for wireless data transmission in order to be compatible with mobile computing devices. Several semiconductor IC manufacturers offer Bluetooth low energy (BLE) and Wi-Fi system on a chip (SoC) solutions. Two examples are the Nordic Semiconductor nRF52840 BLE SoC (Norway) and the Microchip Technology ATWINC1510 Wi-Fi SoC (USA). The use of wireless protocols like BLE and Wi-Fi in NIRS devices presents several constraints, such as managing power consumption, data throughput and latency, network interference and reliability, and range and coverage limitations. By consuming 1 to 35 mA on average, BLE is low power, making it suitable for battery-powered wearable applications. However, the tradeoff is that BLE may struggle with high data bandwidth requirements and operating distance. In contrast, Wi-Fi supports higher data rates and better range, but has greater sensitivity to interference and greater power draw [62, 63]. Groups have also investigated the use of the user datagram protocol over Wi-Fi rather than the more typical and reliable transmission control protocol to prioritize speed over stability [64]. For untethered operation in free-range settings, onboard data storage (e.g. SD cards or embedded flash memory) enables wearable NIRS devices to buffer measurements locally before wireless transmission or operate fully offline [65]. Commercial systems such as the NIRx NIRSport2 (Germany)[66], Artinis PortaMon and Brite (The Netherlands) [67], and Cortivision Spectrum C23 (Poland) [68] incorporate onboard storage capabilities to support extended monitoring sessions with recording times ranging from 2 to 250 h, which is dependent on application, channel count, sampling rates, and duty cycle.

Power management is important in any wireless battery-powered wearable device to maximize operation time between charging. Power consumption can be managed through duty cycling, sleep modes, on-board processing (e.g. data compression before transmission and downsampling), and choice of wireless protocol. Energy harvesting from body heat or movement has also been explored for wearable devices [69]. Across the reviewed wearable NIRS publications, we found that there is no standardized reporting of battery life under clearly defined settings (optode count, sampling rate, wireless protocol, and processing load), despite many offering qualitative claims of battery duration [70, 71].

#### 3.1.4 Semiconductor ASICs

While discrete component designs—utilizing off-the-shelf ICs and microcontrollers—are often favored for rapid prototyping due to their design flexibility and lower initial cost, they are limited by larger footprints and higher power consumption. These are key design constraints for wearable devices. In contrast, incorporating semiconductor CMOS ASIC technology represents the ultimate stage of system integration for wearable NIRS, offering a path to minimize size, power consumption, and noise. However, the transition from discrete to ASIC-based design involves significant system-level tradeoffs (table 3).

**Table 3.** Comparison of discrete (off-the-shelf) and ASIC-based (custom) implementation strategies for wearable NIRS front-end electronics.

Feature	Discrete (off-the-shelf)	ASIC-based (custom)
Prototyping speed	Fast (weeks)	Slow (months/years)
Design flexibility	High (easy to swap components)	Low (fixed after fabrication)
Up-front cost	Low	Very high (tape-out fees)
Unit cost (volume)	Higher	Lower
Size/footprint	Moderate to large	Ultra-compact (single chip)
Integration level	Limited to PCB level	On-chip (detectors + logic)

*Terms: ASIC—application-specific integrated circuit; PCB—printed circuit board*

Discrete designs allow for iterative modifications and quick time-to-demonstration, while ASICs require highly skilled analog/mixed-signal circuit designers, high up-front non-recurring engineering costs, and long development cycles (often 6–12 months per fabrication tape-out). Despite these barriers, ASICs are indispensable for high-volume manufacturing and for achieving the extreme miniaturization required for wearables. Furthermore, ASICs enable the co-integration of optical detectors (e.g. Si PIN photodiodes, APDs, SPADs, SiPMs) directly with front-end electronics on the same substrate.

As such, ASICs have been designed and optimized for use in wearable NIRS to perform specific functions (e.g. receiver amplification, source control, demodulation), typically requiring less power and space which benefits wearable designs [72]. Beyond the ASIC source drivers already discussed, NIRS-specific ASICs have also been used to improve SNR, increase a TIA’s detectable current range, and minimize power consumption [73]. Several groups have reported the use of ASICs for lock-in detection in CW-NIRS devices as part of the on-board signal-conditioning pipeline to reduce ambient light interference [52, 74, 75].

Power conservation can also be more effectively achieved with ASIC integration. For example, ASIC source drivers can adjust LED intensity and timing based on real-time photodetector feedback, ensuring that the system delivers only the optical power required to maintain a target SNR [72, 76]. Additionally, charge-counting-based light-to-digital converters can replace conventional TIAs and ADCs, integrating sampling and filtering into a single stage to reduce both noise floor and component count [77].

Ultimately, while the manufacturability challenges—including complex testing and packaging of optoelectronic ASICs—remain a hurdle for small research groups, the move toward fully integrated ‘NIRS-on-a-chip’ is essential for the large-scale deployment of clinical-grade continuous monitoring. This is especially true for the more complex TD- and FD-NIRS techniques. For example, in the context of TD-NIRS, the integration of SPAD arrays with on-chip TDCs in modern CMOS technology nodes can result in higher pixel fill factors and energy efficiencies [78]. Later we discuss specific examples of wearable TD- and FD-NIRS systems based on ASICs in sections 4.2 and 4.3, respectively.

#### 3.1.5 Optical coupling

All NIRS devices require secure and consistent optical coupling to the tissue (particularly the detector channels) for accurate measurements and robustness to motion. While the use of wearable probes that utilize fiber optics to transfer light between tissue and optical components are common for stationary NIRS devices and some ‘backpack style’ devices [30, 79], fully integrated wearable devices will place sources and detectors directly at the tissue interface for compactness [80]. Direct coupling reduces the overall size profile and can reduce motion sensitivity by eliminating fiber tugging. Modular, fiber-free, and wearable high density (WHD) diffuse optical tomography (DOT) battery powered systems have been designed to be robust to motion, with one WHD system achieving substantially higher decoding accuracy compared to a traditional fiber-based DOT system (99.3% versus 37.9%) [47, 81]. Spring-loaded optodes in tightly fitting headware are widely used to maintain consistent scalp contact and reduce motion-induced coupling changes. Although not explicitly stated, the aforementioned accuracy improvements may also be a result of a design architecture consisting of fiber-free spring-loaded optodes and cinching mechanisms that allows the 3D-printed cap to better mold and conform to different head shapes while creating a tight, comfortable fit [47, 52, 81–86].

Brain-wearable NIRS systems should also account for hair-related optical attenuation and coupling variability during both probe design and data quality control filtering. Hair, especially on the head, introduces significant challenges for optical coupling. Hair density, color, and coarseness can attenuate detected intensity, increase variability, physically prevent optodes from contacting the scalp, and introduce additional scattering paths [82, 87, 88]. Koizumi *et al* reported that conventional optodes (i.e. sources and detectors with no light guides) were less effective in the presence of hair and noted that attaching a 1 mm fiber optic to the proximal end of the optodes improved light delivery [89]. Similarly, a study reported a significant SNR increase of 10 dB during detected brain activations using ‘brush optodes’ (bundles of 250 *µ*m optical fibers that attach to commercial functional NIRS (fNIRS) optodes) that comb through hair to increase scalp coupling. More recently, hair clearance devices for a commercial NIRS optode demonstrated a significant 18 dB rise in SNR compared to when traditional hair parting techniques were performed [85]. Furthermore, the group designed attachments for all hair types and densities to improve inclusivity.

### 3.2 Multiplexing strategies

As the number of optodes required increases, such as those required for high-density fNIRS and whole-head fNIRS configurations, wearable NIRS designs are challenged with additional hardware complexity, reductions in sampling rates, and the potential for inter-channel crosstalk. Multiplexing schemes including time-division multiplexing (TDM) [46, 71, 90], spatial-division multiplexing (SDM) [91, 92], and frequency-division multiplexing (FDM) [54] can address these constraints, each with distinct tradeoffs that can become consequential as the channel count and density increases.

TDM involves sequential activation of sources to minimize inter-channel interference (optical and electrical), reduce average power consumption and system heating, and simplify hardware design [52]. However, the resulting overall frame rate scales inversely with the optode count, constraining the viability of TDM as a standalone approach. SDM circumvents the frame rate limitation by simultaneously illuminating spatially separated source groups, but imposes geometry-dependent constraints: tissue light attenuation between simultaneously activated groups must be sufficient to prevent optical crosstalk, with one study suggesting 90 mm between nearest simultaneously illuminated sources [93]. FDM can achieve higher sampling rates than TDM by activating sources simultaneously at different carrier frequencies, but this shifts inter-channel crosstalk from the optical to the spectral domain and requires additional complexity for demodulation [94].

Application constraints (e.g. optode count, required sampling, and power consumption) drive which choice of multiplexing is appropriate, and even all three can be applied simultaneously. To manage the sequencing and demodulation demands of these combined approaches, FPGAs are particularly well-suited, as programmable logic can implement custom rapid sequencing, parallel demodulation, and provide real-time on-board data processing at high sampling rates [42, 57, 58]. A related approach is to use adaptive sampling strategies such as binary neural network-driven channel selection [92] to activate only the source-detector pairs relevant to a user’s current brain state, reducing energy consumption without sacrificing functional sensitivity.

### 3.3 Wearability

In addition to high technical performance (e.g. accuracy, precision, stability, speed, and battery life), wearable NIRS devices must be designed with user-centric factors to ensure they are practical and effective for long-term monitoring. Some crucial aspects include comfort (e.g. compact and lightweight, flexible), safety (e.g. no high voltages, safe operating temperature, biocompatible/hypoallergenic skin interfaces), and durability (e.g. withstand sweat, humidity, and wide operating temperatures) [95, 96]. Devices typically use flexible materials (e.g. flexible PCBs, elastic bands, neoprene head caps) to comfortably maintain stable optical coupling and SNR across various body shapes, head sizes, and motion [97–102]. Additionally, because these devices are meant for prolonged use, minimizing irritation at the sensor-tissue interface is critical, especially when designed for populations with sensitive skin such as neonates. Recent progress in soft, thin, skin-conformable flexible electronic substrates are particularly promising for creating low-profile comfortable sensors and including optoelectronic components such as photodetectors [103] and microLEDs. For example, Rwei *et al* demonstrated a flexible wireless cerebral hemodynamic monitoring device designed for pediatric use composed of silicone encapsulated ultrathin copper-coated $25\,\mu$m polyimide PCB substrates [104].

Wearability also includes the device’s ability to withstand repeated use and diverse environmental conditions. The materials used should avoid skin irritation and allergic reactions, ensuring user comfort and safety during prolonged use [105]. More importantly, careful electrical insulation and appropriate voltage components should be selected to enhance safety and reduce the need for bulky power converters. For NIRS devices with multiple channels, such as in fNIRS, modular designs allow for easy customization and adjustment, making devices suitable for various applications (e.g. motor, visual, auditory only experiments) and populations [106]. Modularity also facilitates more manageable maintenance and upgrades.

### 3.4 Motion

Although NIRS is less susceptible to MAs than other imaging modalities (e.g. electroencephalography-EEG and noncontact imaging such as MRI), wearable NIRS devices remain sensitive to MAs as the user freely moves. A primary source of MA signal contamination is due to changes in optical coupling to the tissue, which can be mitigated with the skin interface hardware designs discussed in section 3.1.5. In addition, physiological MAs can arise from systemic hemodynamic responses to movement, including vasomotor activity and changes in blood pressure that can produce spurious signals that are indistinguishable from typical hemodynamic processes [107]. As such, physiological sources of MAs are unavoidable since they are inherently present in the detected signal. Signal processing methods to identify and eliminate MAs are an important and active area of research aimed at improving data quality, which we discuss in this section.

Comparative studies have concluded, unsurprisingly, that motion correction is preferred to data rejection alone, although performance depends on the MA type, experimental design, and the degree to which the algorithm preserves the underlying signals [108–111]. Common MA removal strategies include wavelet-based methods [110, 112–114], variants of principle component analysis [115], spline interpolation [116, 117], discrete Kalman filter [118], and correlation-based signal improvement (CBSI) [119]. Zhou *et al* introduced a dual-stage MA removal algorithm that explicitly classifies MAs as high-spike, low-spike, and baseline shifts before correction, unlike many traditional MA methods that do not explicitly differentiate between artifact types, but instead apply a single correction strategy across detected events [117]. This method uses an artifact-aware, coarse-to-fine correction pipeline coupled with task-level classification-based evaluation, demonstrating that MA suppression can be optimized to preserve functional decoding performance in wearable fNIRS. Although, due to the two stage processing and two-sided moving standard deviation window, it is fundamentally an offline processing method and less suited for real-time use. Methods that are generally feasible in real-time scenarios are CBSI, the discrete Kalman filter, and threshold-based wavelet due to their causal, low-latency architectures. Each approach, however, carries tradeoffs rooted in oversimplified MA assumptions. For example, CBSI’s reliance on HbO/HHb anti-correlation risks overcorrection when those assumptions break down. Machine learning (ML) based techniques, including support vector machines (SVMs) [57], artificial neural networks (ANNs) [120], and deep learning (DL) [121, 122], have been explored in recent years. One work utilizing a denoising autoencoder outperformed all previously mentioned strategies and achieved 100% accuracy in removing MAs [121].

Hardware motion sensors, such as the STMicroelectronics ISM330DHCX (Switzerland) and Bosch BMI270 (Germany), can help identify potential MAs. To this effect, some NIRS devices have integrated inertial measurement units (IMUs)–accelerometer, gyroscope, magnetometer, or a combination of the three–to identify motion events [123–126]. In one of the earliest implementations in a wearable NIRS device, Kim *et al* built a wireless 12-channel CW-fNIRS device equipped with an accelerometer for MA detection [124]. With the use of an adaptive noise cancellation algorithm [127], transient MAs were successfully removed while keeping normal fluctuations uncorrelated with head movement. Siddiquee *et al* developed a custom wearable two-channel CW-NIRS system with an integrated IMU sensor (containing an accelerometer, gyroscope, and magnetometer) to record NIRS signals alongside movement artifacts [125]. The corresponding IMU data were used to estimate and remove artifacts from the NIRS signals using an autoregressive with exogenous input (ARX) method. The ARX method predicts future values based on past values and external inputs. Tested on four healthy subjects, using all three IMU sensors significantly improved SNR (∼5–11 dB) and artifact removal compared to using the accelerometer alone, highlighting the potential of multi-axis rotational information for wearable MA correction.

It is worth noting that sensitivity to motion may not be uniform across NIRS modalities. CW systems are the most vulnerable to optode-coupling artifacts given their reliance on raw light intensity [109]. FD systems can offer greater resilience as the phase slope, measured across multiple SDS, is less sensitive to optode-level coupling offsets and superficial tissue artifacts [128, 129]. TD-NIRS instruments, by resolving the photon DTOF, exhibit greater robustness to physical optode displacement and systemic blood pressure oscillations [30, 130]. However, we could not identify any studies that provide a comprehensive comparison between the three approaches in their ability to mitigate hardware and physiological MAs. The field could benefit from such an analysis.

## 4 Wearable NIRS devices

Now that we have discussed design considerations that are unique to wearable wireless NIRS devices, we present a comprehensive review of recent advancements in wearable NIRS technologies and implementations, organized by measurement approach (CW-, TD-, and FD-NIRS). A detailed listing of the wearable NIRS devices covered in this review and their design and performance characteristics is provided in the Supplementary Material.

### 4.1 CW-NIRS devices

**Figure 3.**
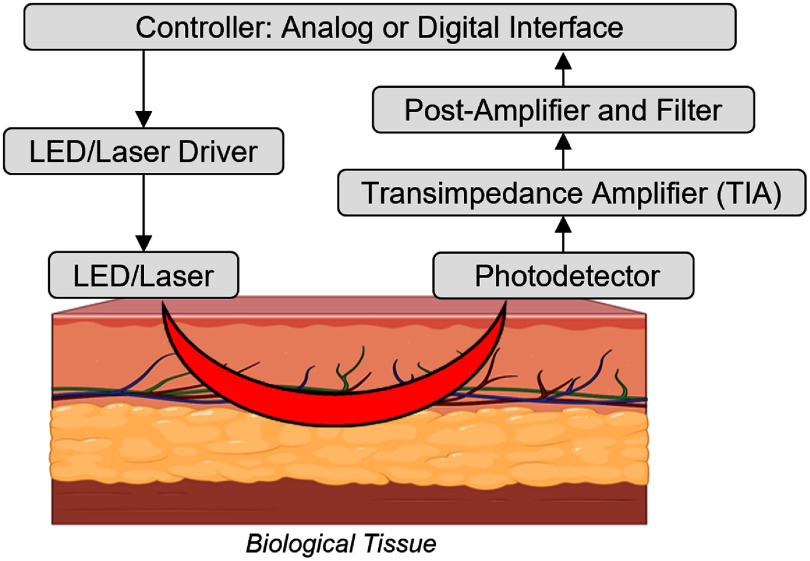
Architecture of a CW-NIRS system. A central controller coordinates the measurement sequence, including the activation of optical source drivers (e.g. setting the current) to deliver light into the tissue. Remitted light is captured by a photodetector, whose signal is subsequently amplified and filtered for chromophore analysis. While a single SDS is depicted, the architecture can be scaled to multichannel configurations through spatial, frequency, or time-division multiplexing.

A generalized block diagram of a CW-NIRS system is shown in figure 3. LED or laser source light is delivered into tissue, a photodetector captures the diffusive light, typically in reflection geometry, and the resulting photocurrent is converted to an amplified voltage using a TIA before amplification, filtering, and ADC. Following this hardware architecture, the specific implementation of CW-NIRS varies significantly depending on the target tissue. We therefore review CW-NIRS wearable systems by their application site: first, we review neurosensing devices worn on the human head, followed by a discussion of systems optimized for other applications such as peripheral skeletal muscle.

#### 4.1.1 CW-fNIRS systems

The largest and most developed category of wearable NIRS instruments is for fNIRS. Mostly utilizing CW-NIRS, fNIRS probes neural activity by measuring changes in detected light intensity across one or more SDS and range from single channel implementations [131] to HD configurations consisting of up to thousands of channels [47, 81]. A defining advantage of wearable fNIRS systems is their ability to provide neurosensing and neuroimaging in naturalistic settings, a feature not available with gold-standard neuroimaging modalities such as functional MRI. Wearable fNIRS systems are therefore a powerful tool for neuroscience and the demand for high-quality imaging drives continued innovation in compact, low-power, high optode density, and mechanically robust systems.

To organize our review of these devices, we classified wearable CW-fNIRS systems into three categories: devices developed in research labs for the prefrontal cortex (PFC) that are used on the forehead, research devices based on a cap design with a wide field of view that covers the crown and/or the side and back of the head, and devices that have reached the commercialization stage.

*Wearable CW-fNIRS systems for the PFC*

The PFC is a common target for wearable CW-fNIRS due to the lack of dense, coarse hair on the forehead, which provides good optical coupling, higher SNR, and more repeatable measurements compared to other regions of the brain. This reduces the need for complex hair-mitigation strategies as well as the need for more sophisticated hardware designs such that CW-fNIRS for the PFC can be developed with simpler and lower-cost system architectures. As a result, several lightweight, low cost PFC-based CW-fNIRS systems have been reported. For example, dual wavelength systems with 10-channel [38] and 1-channel [131] configurations with wireless communication and total device weights of ∼100 g and 150 g, respectively, were created with material costs of ∼$200. Similar forehead-mounted wearable designs have been reported with variations in hardware configuration and channel count [132, 133]. While these systems reduce hardware complexity and cost, these designs have limited coverage area measuring only the PFC and are well suited only for examining higher level executive function. Consequently, such devices should not be used in functional connectivity, motor rehabilitation, and visual- and auditory-driven studies.

**Figure 4.**
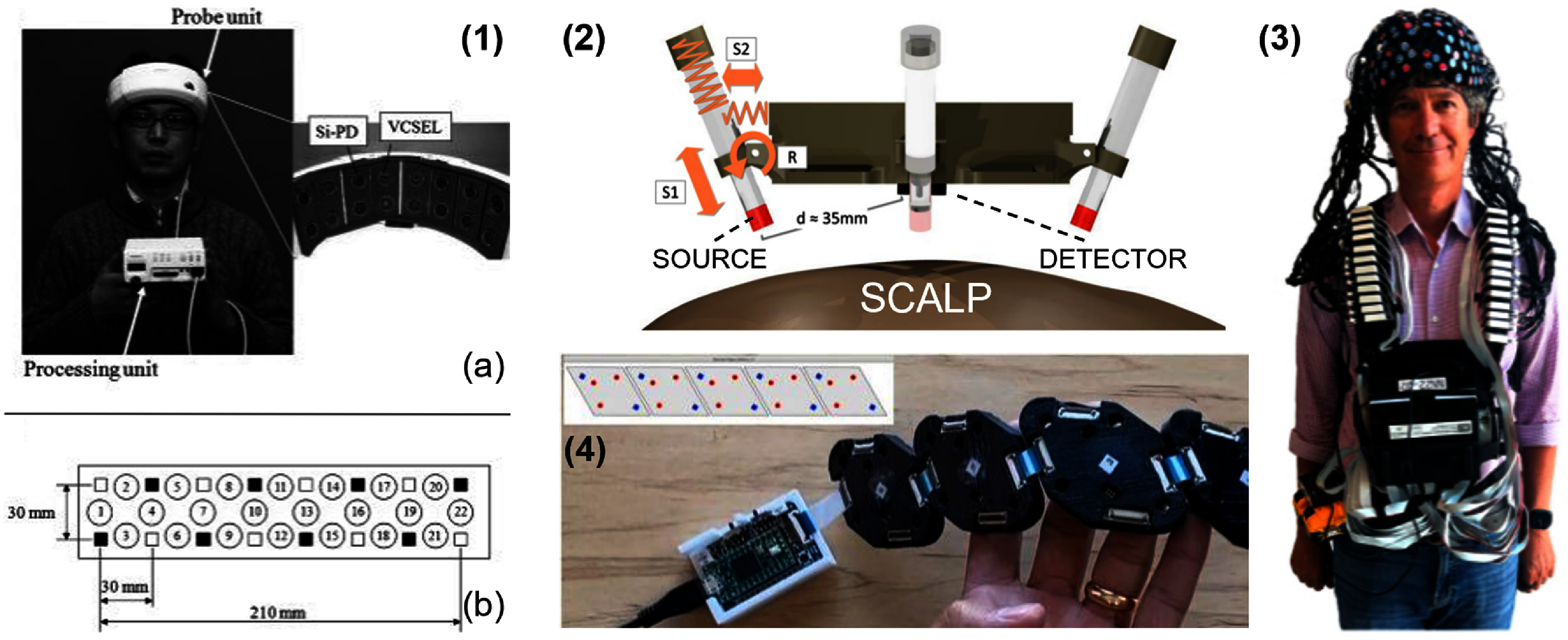
(1) Wearable CW-fNIRS system and technology examples. (a) The probe unit in the work by Atsumori *et al* [134] has a flexible pad featuring eight sources and eight detectors connected to a body-worn processing unit via flexible cables. (b) Its probe layout including VCSELs, Si-PDs, and 22 measurement channels (indicated by number), each separated by 30 mm. Reprinted from [134], with the permission of AIP Publishing. (2) Spring loaded assembly for brain measurement from von Lühmann [52] where spring S1 is for coupling and alignment of the LED and spring S2 and rotatory joint R is for maintaining perpendicularity between LED and scalp. The SDS (d) is 35 mm. Reproduced from [52]. CC BY 4.0. (3) Open-source, high density ninjaNIRS22 system with 200 optodes by O’Brien *et al* [58] shown being worn by participant. Reprinted with permission from [58] © The Optical Society. (4) Spatially aware flexible, modular NIRS imager by Xu *et al* [58] and automatic optode placement in space based on module-to-module connections, shown in top-left illustration with LEDs in red and photodiodes in blue. Reproduced from [91]. CC BY 4.0.

Ensuring stable and efficient optical coupling between optodes and the scalp is critical for high-quality measurements, particularly as optode count and size/mass of onboard electronics increase. Added device weight can degrade coupling efficiency and therefore increase measurement variability if not properly managed. One of the earliest full-forehead implementations addressing this challenge is shown in figure 4(1), featuring a 22-channel, dual-wavelength configuration weighing approximately 400 g with adjustable headgear to maintain consistent contact between optode and tissue [134]. Subsequent implementations in the form of spring-loaded optode assemblies [52] have further improved measurement reliability by mechanically accounting for head curvature and motion, as illustrated in figure 4(2). Spring-loaded optodes improve coupling, but the design introduces additional mechanical complexity and components leading to increased fabrication cost and system weight that could reduce subject comfortability over prolonged use.

To be used effectively in real-world situations, especially outdoors, fNIRS systems must effectively suppress ambient light, which is typically achieved through low complexity solutions such as shading caps [135], light blocking headbands [131], or opaque optode encasements, as shown in figure 4(1) [134]. The entire wearable system can be covered in neoprene caps, rigid helmets, or black 3D printed housings. These approaches are commonly employed in wearable cap-based systems and the commercial devices described in the next paragraph and at the end of this section, respectively.

*Wearable cap-based CW-fNIRS systems*

CW-fNIRS systems designed for larger cortical coverage, including multiple regions, typically rely on wearable conformable head caps to secure optodes across the scalp and are less likely to be fully fiber-free than forehead-only designs. An early device–claimed to be the first untethered device used during outdoor activities–included a fabric-based cap to secure eight dual-wavelength (760 and 850 nm) LED sources and eight detectors to the head [37]. The cap employs 12 mm long plastic optical fibers to guide light from the scalp to the photodiodes and the system employed time and frequency (intensity-modulation) multiplexing. The data acquisition board was housed in a backpack, with a USB connection to a laptop computer that provided data transfer and power for up to two hours of continuous operation. This design was later commercialized, becoming the NIRx NIRsport device [136]. Subsequent cap designs have expanded on this architecture by adding additional wavelengths, sources, detectors, and onboard processing capabilities [60, 137]. While this system employed effective cable management techniques, the required wires and backpack, or waist-mounted holder in newer models, for the controller may be cumbersome for subjects engaging in naturalistic studies such as exercise-based protocols.

Whole-head coverage is often achieved using modular approaches. In these designs, sources and detectors are packaged in discrete modules that can be assembled to obtain the desired head coverage [58, 61, 75, 91, 106, 138–140]. From an engineering perspective, this modular approach allows for the easy customization of optode positioning and illumination sequencing for different study objectives and head geometries while simplifying maintenance. However, several foundational modular designs—including large-scale systems with over 1000 NIRS channels per wavelength [140], palm-sized modules with up to 41 dual-wavelength sources and 39 detectors [138], and fiber-less DOT systems with multi-wavelength LED assemblies sensitive to cerebral cytochrome-c-oxidase activity [61, 141]—rely on tethered PC connections for power and data transmission, placing them outside the scope of this review. Nevertheless, these implementations established the feasibility of modular architectures and motivated subsequent wireless and battery-powered designs.

Wyser *et al* pursued a modular approach, prioritizing per-module sensitivity over channel count by integrating SiPMs and four-wavelength LEDs into miniaturized optode tiles (size 20.5 × 18 × 8 mm, weight ∼5 g) that enabled short-channel regression to suppress physiological interference, improved measurement robustness, and achieved a dynamic range of $ {\gt} 160$ dB [106]. This system has since been commercialized as the optoHIVE system, a cap-based architecture in which all the optodes are housed inside plastic enclosures that can be utilized in a plug-n-play format [142]. Building on this architecture, the ninjaNIRS system [58], shown in figure 4(3), represents a fully wireless and battery-powered modular approach housed within a custom open-source 3D-printed cap fabricated from thermoplastic urethane (TPU) [139]. The flexible TPU material conforms to diverse head geometries, while an open mesh design facilitates hair parting and scalp access without compromising structural integrity. The system itself utilizes separate source and detector modules featuring dual-wavelength (730 and 850 nm) illumination and photodiodes with integrated TIAs [58]. The system, now in its third generation, is scalable with as few as 2 or up to 200 optodes for HD-DOT. Modular architectures provide users with the freedom to design protocols specific to their interests and can reduce the time spent troubleshooting and repairing systems. However, these benefits come at the risk of increased system complexity, higher engineering labor and costs, and reduced sampling rates for larger SD arrays.

Beyond discrete module assemblies, flexible PCBs offer a complementary approach for linking modules in wearable NIRS devices across the curved head, enabling compact and lightweight integration. Their inherent flexibility enhances device comfort while ensuring reliable electrical connections and signal integrity. A flexible diamond-shaped modular optical brain imaging (MOBI) system demonstrated this approach, in which individual flexible modules containing three dual-wavelength sources and two detectors are interconnected via diamond-patterned flexible PCBs, as shown in figure 4(4) [91, 143]. This approach showcased flexible, spatially aware modules with IMUs for real-time monitoring of optode motion for robust MA rejection. Notably, MOBI modules are also compatible with the ninjaNIRS platform described above, enabling integration into the open-source ninjaCap architecture. In its full head configuration, however, the architecture is estimated to achieve a data acquisition rate of 2.4 Hz, which falls below the threshold needed to reliably capture pulsatile signals at typical resting heart rates, and presents challenges for computing scalp coupling index, a metric used to assess probe-to-tissue contact quality.

*Commercial CW-fNIRS devices*

Several wearable CW-fNIRS devices are commercially available, primarily designed for clinical research applications, and feature designs that emphasize comfort, robustness, ease of use, and reproducibility. Typical designs include dual-wavelength illumination, wireless data transmission via Bluetooth or Wi-Fi, integrated IMUs for motion tracking, rechargeable batteries, built-in short separation channels, and real-time recording at sampling rates ranging from 10–250 Hz [66–68, 142, 144, 145]. A practical distinction between research-grade and commercial fNIRS systems is the inclusion of validated software for real-time data visualization, processing, and analysis. In the remainder of this section, we highlight a few wearable CW-fNIRS systems currently on the market. Readers can find a detailed table of commercially available wearable wireless fNIRS systems in an online database [146].

**Figure 5.**
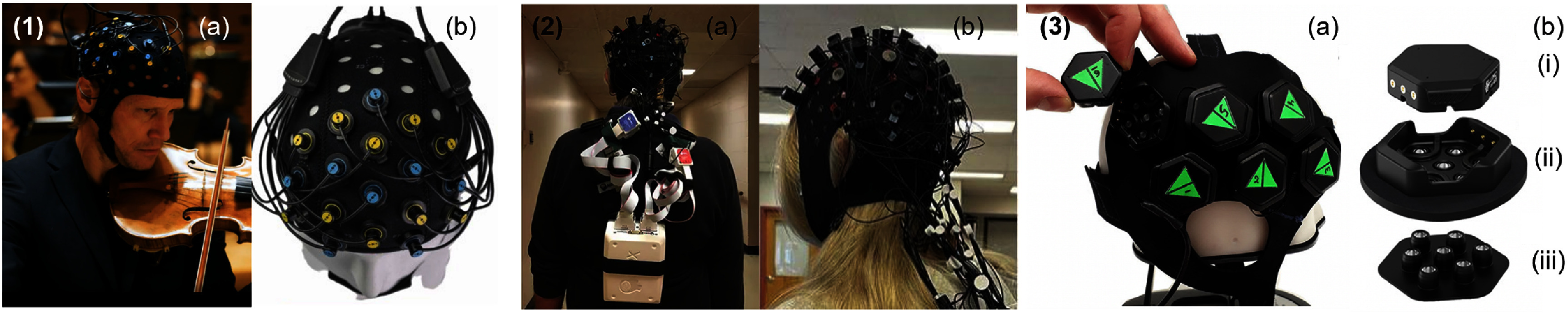
Images of a selection of commercial CW-fNIRS systems. (1) Artinis Brite head cap system (a) worn over the right hemisphere by a first violinist shown in Fagerland *et al* [147]. Reproduced from [147]. CC BY 4.0, and (b) a top view of the device by Martins *et al* [148]. Reproduced from [148]. CC BY 4.0. (2) Image of NIRx NIRsport2 in a (a) backpack configuration being worn by subject as shown by Lim *et al* [149]. Reproduced from [149]. CC BY 4.0. and (b) side view of the head cap reported by Benam *et al* [150]. Reprinted from [150], Copyright (2025), with permission from Elsevier. (3) (a) Gowerlabs LUMO HD-DOT device that is integrated with (b)(ii) docks in which the (i) hexagonal tiles containing the optoelectronic components snap. (iii) Its light-guide attachment contains seven optical fibers 4.5 mm in length as described by Frijia *et al* [101]. Reproduced from [101]. CC BY 4.0.

Artinis Medical Systems offers several wearable fNIRS instruments with channel counts ranging from 6 to more than 100, with sampling rates of up to 150 Hz for low-optode configurations. Larger channel count systems, as shown in figure 5(1), are integrated into a neoprene cap weighing approximately 300 g [67], with dual-wavelength LEDs centered near 760 nm and 850 nm, although additional wavelengths are available upon request. Data can be transmitted wirelessly via Bluetooth or stored locally for at least 100 h for offline post-processing and analysis. All circuitry is housed in a control unit mounted at the rear of the cap or a body worn strap. Cap-based configurations are compatible with EEG and transcranial electrical stimulation, enabling multimodal acquisition and closed-loop experimental paradigms. Additional features include synchronized hyperscanning for multi-subject studies, proprietary software for real-time data processing and visualization, cap sizes spanning infant to adult populations, 6-axis IMU for MA rejection, and dedicated transport case. Notably, Artinis MediBrite is the first portable European NIRS device certified for clinical use under Medical Device Regulation (EU) 2017/745.

Using a similar cap-based architecture, the NIRx NIRSport2 device features spring-loaded grommets and variable tension spring holders for optimal scalp coupling and comfort [66]. Depicted in figure 5(2), the system is scalable from 8 to 80 sources and detectors, allowing up to 256 measurement channels, and sampling rates up to 240 Hz. In contrast to Artinis systems, NIRSport2 also includes 9-axis IMUs at the probe-level (accelerometer, gyroscope, and magnetometer) to support detailed motion tracking. However, the NIRSport2 is significantly heavier, weighing approximately 900 g. NIRx provides proprietary acquisition and analysis software and has partnered with Brain Innovation (The Netherlands) to offer their users real-time brain-computer interface (BCI) and neurofeedback capabilities through the Turbo-Satori platform. This includes online statistical analysis tools such as SVMs, general linear models (GLM), frequency spectograms, short-channel regression, and artifact removal [151].

Gowerlabs’ (United Kingdom) LUMO system is notable for its modular optode design that provides HD full head CW-fNIRS measurements [144]. Shown in figure 5(3), the core sensing element is a hexagonal module, or ‘tile’, which integrates three sources, four detectors, and an IMU. The tiles are attached to a wearable cap and interconnected through flexible circuitry that accommodates variations in head shape and size. Two tiles can form up to 48 channels and additional tiles can be added to expand head coverage. Integrated optical filters and light guides minimize ambient light interference and help maintain stable contact with the scalp, while optional spring-loaded light guides support measurements across various cortical regions. The LUMO system is available in cap sizes ranging from infant to adult, allowing coverage from localized cortical areas to the entire head, and its modular construction facilitates cleaning, reconfiguration, and reuse across subjects. We note that a wireless battery-powered LUMO device is currently under development and is not available to the public. The LUMO has been demonstrated to operate under battery power for untethered wireless operation [99].

#### 4.1.2 Wearable CW-NIRS devices for tissue oximetry

There has been significant development of wearable CW-NIRS devices for applications beyond fNIRS. This includes tissue and cerebral oximetry, which we classify separately because they tend to have a smaller size profile and fewer channel counts than fNIRS systems. Systems have also been developed to monitor muscle physiology and even deeper tissues such as the placenta.

Cerebral oximetry represents one of the most intensively researched use cases of CW-NIRS, and it is currently used during pediatric critical care and cardiac surgery for both children and adults [152, 153]. Since in-patient care does not require wireless operation, most of the devices developed for this purpose do not fit the definition of wireless NIRS. However, there are many outpatient and wellness applications such as during exercise or for ambulatory monitoring. As with fNIRS systems, flexibility and adaptability at the skin interface is an important attribute for wearable cerebral oximeters. One device addressing these constraints featured a soft, skin-interface device using medical-grade silicone and an adhesive [104]. The device circuitry consisted of four copper layers on a flexible PCB with a replaceable magnetically coupled battery, enabling extended operation without device removal and improving safety during long-term monitoring. Similar work has been reported, but it has been adapted for adult populations where a larger SDS is required. Wu *et al* developed an open-source modular FlexNIRS platform that enables a self-calibrated multi-distance approach for measuring cerebral tissue oxygen saturation at 100 Hz [154]. Cerebral oximeters primarily focus on oxygenation in the PFC to avoid hair interference and increase SNR; however, this one cortical region is not indicative of cerebral health as a whole and desaturation or ischemic events in posterior regions may go unnoticed [155].

**Figure 6.**
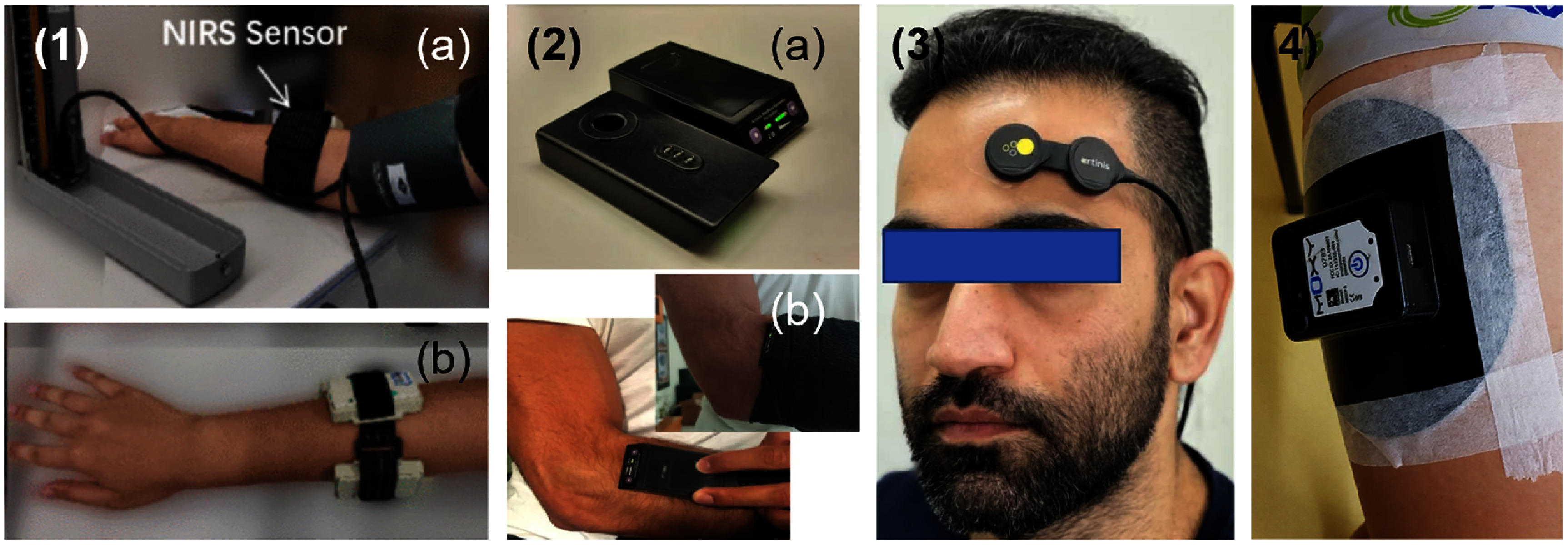
Examples of prototype and commercially available tissue oximeters. (1) Xie *et al* [157] reported this (a) research grade SmO_2_ sensor evaluating brachioradialis muscle oxygenation during an occlusion test and (b) four sensors (two shown) worn on the forearm during a hand motion experiment, © (2022) IEEE. Reprinted, with permission, from [157]. (2)(a) Top and bottom view of Artinis PortaMon and (b) device attached on a subject’s forearm with a black bandage as published by Cortese *et al* [160]. Reproduced from [160]. CC BY 4.0. (3)(a) Image of Artinis PortaLite MKII adhered to the forehead of a subject by Hakimi *et al* [161]. Reproduced from [161]. CC BY 4.0. (4) Moxy Oxygen Monitor sensor with sunlight blocking guard worn on cyclist’s vastus lateralis as described by Crum *et al* [162]. Used with permission of John Wiley & Sons—Books, from; permission conveyed through Copyright Clearance Center, Inc. [162] John Wiley & Sons. © European College of Sport Science.

Wearable CW-NIRS architectures have been developed for monitoring skeletal muscle oxygenation (SmO_2_), typically at the limbs for use during exercise and training. Myoglobin is an oxygen binding and storing protein in muscles whose optical absorption is indistinguishable from hemoglobin with the few wavelengths typically found in wearable devices. NIRS devices designed for sensing SmO_2_ report a combination of hemoglobin and myoglobin concentrations which represent a collective measure of both oxygen delivery to the microvasculature and oxygen storage status within myocytes, rather than a purely vascular metric. Akin to cerebral oximeters, wearable SmO_2_ NIRS sensors tend to be more compact than fNIRS systems, as a large field of view coverage is usually not required. However, muscle synergy theory suggests that coordinated movement arises from the interaction of muscle groups [156], motivating the use of multiple wearable sensors to capture this activity. For example, Xie *et al* created four custom wearable wireless NIRS devices with triple-wavelength sources that transmitted data to a custom Bluetooth receiver to capture rehabilitation-related movement from each sensor simultaneously (figure 6(1)) [157]. However, multichannel sensing in close proximity introduces significant risks of optical crosstalk, where light from one sensor is erroneously captured by the detector of another. Mitigation of this interference remains a design challenge that could be addressed by the multiplexing strategies discussed in section 3.2. Choi *et al* produced an 8-channel wearable NIRS sensor with the intention of mapping hemodynamic activity via 2D tomography which could be used to study metabolic disorders for those with neuromuscular dysfunction [158]. Although this research was conducted in OBELAB’s research facilities and initial results were favorable, the device has yet to become available commercially.

Due to its low safety risk and accessibility, NIRS sensing is particularly well-suited for applications in maternal and neonatal health. Nguyen *et al* developed a multimodal wearable wireless CW-NIRS sensor for monitoring placental oxygenation transabdominally using three wavelengths and a wide range of SDS from 10–60 mm for a depth sensitivity of up to 25 mm [159]. The lightweight system (∼61 g) incorporated an accelerometer to monitor fetus-induced movements and uterine contractions, and had a window for simultaneous ultrasound imaging. While this represents a significant step in mobile monitoring of maternal-fetal health, the study only examined subjects with anterior and fundal placentas excluding those positioned laterally, posteriorly, and near or on the cervix (i.e. placenta previa). Given the challenges of deep tissue light penetration, attenuation in the uterine muscle, and heterogeneous tissue structure, questions remain about the use of this approach in diverse obstetric populations.

*Commercial devices*

Commercially available wearable NIRS devices exist for monitoring cerebral, muscle, and oxygenation in other tissues. Although these devices share the common goal of enabling tissue oximetry outside of laboratory settings, they each represent a distinct position among core design tradeoffs, specifically the balance between form factor, measurement versatility, and coupling stability during dynamic movement. We illustrate this by discussing two SmO_2_ products that appear frequently in literature and have been directly compared in muscle oxygenation studies [163, 164]–the PortaMon (Artinis) [67] and Moxy (Fortiori Design LLC, USA) [165]– and one multipurpose oximeter that permits monitoring of cerebral and muscle oxygenation in naturalistic studies. For a comprehensive list of commercially available muscle and cerebral oximeters, we direct readers to [146, 163].

Artinis has several devices capable of capturing tissue oxygenation from various anatomical regions. The PortaMon, shown in figure 6(2), is specifically designed with sports science applications in mind. The device features dual-wavelength illumination (760 and 850 nm) with three short SDSs (10–20 mm) that enable short-channel regression to suppress adipose tissue contributions and Bluetooth to enable wireless data transmission. Compared to other Artinis products, the PortaMon prioritizes a compact, lightweight self-contained form factor (∼30 g) over battery life (6 h at 100 Hz sampling rate) and onboard storage (up to 100 h).

For applications requiring greater versatility, the PortaLite MKII is designed for both cerebral and muscle oximetry (figure 6(3)). It is one of the only multipurpose tissue oxygenation devices on the market. While offering more onboard memory capacity (250 h) and battery life (30 h) compared to PortaMon, this extended capability comes with a wearability tradeoff: the PortaLite employs a tethered architecture with a dedicated cable connecting the sensor head to a separate control unit, resulting in a total system weight of ∼240 g. Both devices can be used simultaneously with Artinis fNIRS products, with wireless data synchronization managed through their proprietary OxySoft software.

Moxy Oxygen Monitor, as shown in figure 6(4), represents an alternative muscle oximeter with a distinct design philosophy that emphasizes integration into athletic wear. The device employs four LEDs (680, 720, 760, 800 nm) with two detectors at SDS of 12.5 and 25 mm [164], and operates up to 7 h in low power mode with local data store capacity of ∼5 h. Notably, Moxy has developed sunlight blocking guards and sport-specific garments–including compression shorts and sleeves, running shorts, and cycling bibs–that conform to quadricep and tricep muscle regions, improving optical coupling stability. These design considerations inherently restrict measurements to predefined anatomical sites and as a result, limit the scope for which these devices can be deployed.

### 4.2 TD-NIRS devices

**Figure 7.**
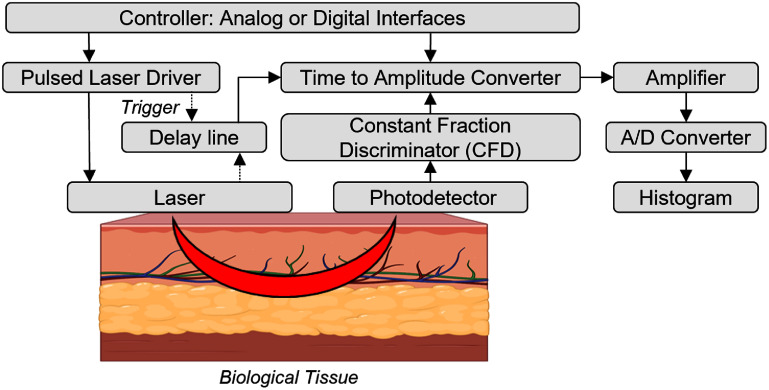
Architecture of a generic TD-NIRS system utilizing time-correlated single photon counting (TCSPC). A central controller coordinates the measurement sequence, signaling the laser driver to deliver short pulses of light into the tissue while simultaneously initiating a timer trigger. A delay line ensures temporal synchronization between the trigger and photon detection window. Detected pulses are timed using a CFD and processed through the TCSPC electronics chain to produce histograms of photon arrival times, which constitute the TPSF. (A/D converter: analog to digital converter). Reproduced from [14]. CC BY 4.0.

The architecture of a TD-NIRS device is more complex than CW-NIRS, requiring fast pulsed lasers, sensitive photodetectors, and typically TCSPC electronics for precise timing resolution as illustrated in figure 7. A standard TCSPC instrument employs a photodetector to identify single-photon events, a constant fraction discriminator (CFD) that generates timing signals by triggering at a fixed fraction of the pulse’s peak amplitude to mitigate pulse-height-induced timing jitter, and a time-to-amplitude converter (TAC) that converts photon arrival time differences into voltage pulses [14, 33]. These pulses are then amplified and digitized by an amplifier and A/D converter, respectively, and stored as histograms representing photon arrival times. Implementing these components requires space, and as such, most TD-NIRS devices are restricted to use in research laboratories [14, 166, 167]. However, TD-NIRS provides a rich set of data that is considered the ‘gold-standard’ in NIRS measurements, so there has been significant effort to make these systems more accessible. In particular, ASIC technology has emerged as a key approach for TD-NIRS miniaturization, similar to CW- and FD-NIRS. In this section, we first detail these efforts and then describe current progress toward wearable and wireless TD-NIRS devices.

#### 4.2.1 Advances in TD-NIRS ASIC technologies

While CW-NIRS systems benefit from hardware simplicity, the transition to wearable TD-NIRS instrumentation requires the radical miniaturization of complex timing and pulsing electronics, a task well suited to ASIC implementation. On the detection side, Conca *et al* developed a highly integrated large area, fast gated, all digital SiPM (8.6 mm^2^ photosensitive area, 37% fill factor) with an on-chip TDC and histogram builder optimized for portable wearable TD-NIRS [168]. The authors claimed this detection architecture as the first to integrate a large programmable active area with fast time gating, effectively replacing bulky timing hardware (that would have required rack-mount instrumentation) with a compact CMOS-based detector capable of sub-nanosecond temporal resolution and high dynamic range. Unfortunately, the SPAD arrays in the fabrication run suffered from a non-uniform breakdown voltage across the detector chip that could cause the pixels to have different temporal responses, timing jitter, and avalanche buildup times leading to performance degradation.

**Figure 8.**
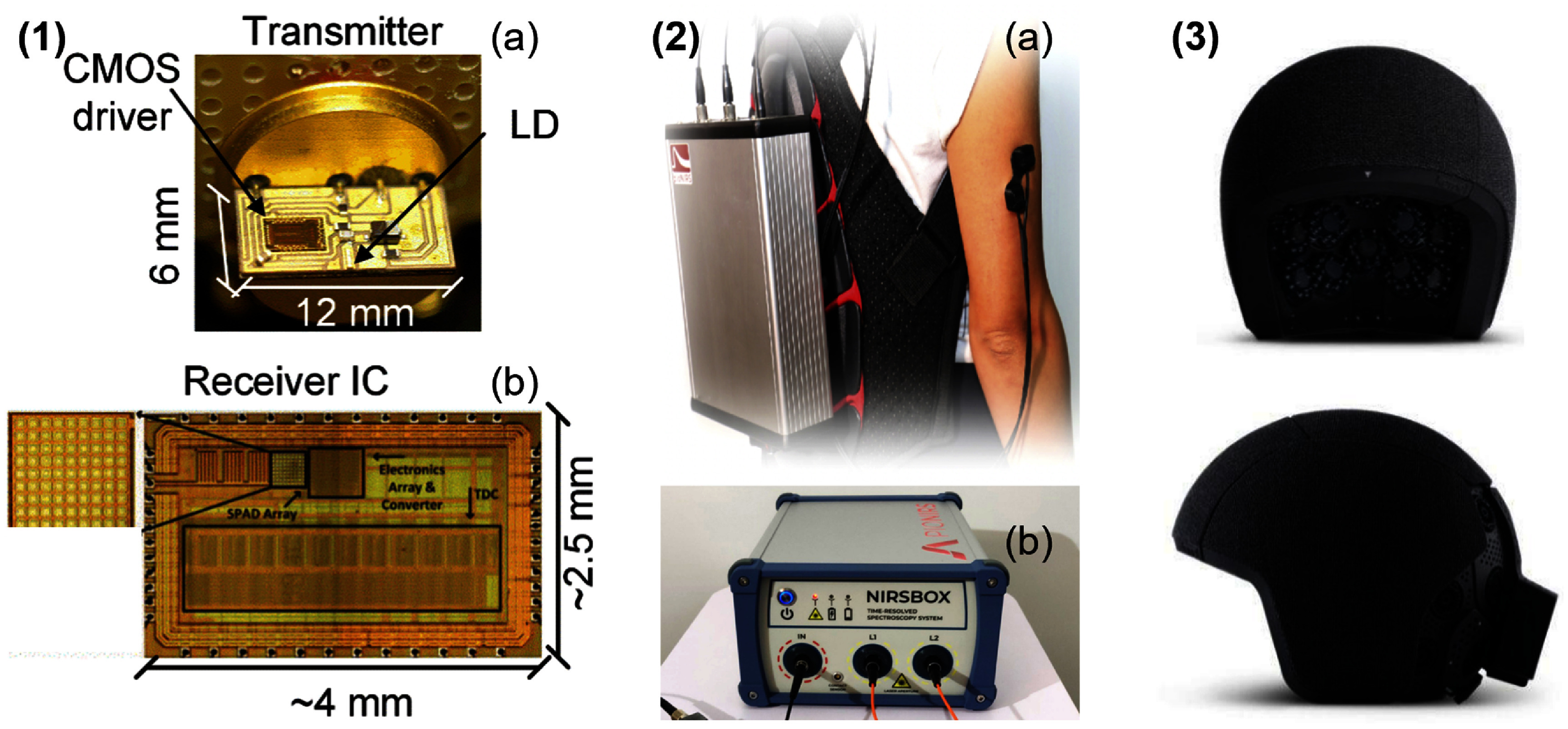
(1) Jansson *et al* [169] reported a CMOS-based TD-NIRS system with (a) an integrated laser driver and a pulsed laser diode transmitter alongside (b) the receiver chip containing a SPAD array and 10-channel TDC, © (2020) IEEE. Reprinted, with permission, from [169]. (2) (a) Lacerenza *et al* [30] reported this research-based TD-NIRS instrument shown worn by a subject in a backpack-based format. Reprinted with permission from [30] © The Optical Society. and (b) the modern commercialized iteration of the same instrument (PIONIRS NIRSBOX) described by Lacerenza *et al* [172]. Reproduced with permission from [172]. (3) Chekin *et al* [83] demonstrated the Kernel Flow2 commercial TD-fNIRS helmet. Reproduced from [83]. CC BY 4.0.

While detector integration addresses half of the TD-NIRS system functionality, advances in pulsed light generation are equally important. Sieno *et al* designed a compact laser pulse source, originally developed for time-of-flight range finding, which co-packages an ASIC laser driver with a laser diode operating in enhanced gain-switching mode [31]. Fabricated in a $0.35\,\mu$m high voltage CMOS process, the driver supports 100 ps laser pulsing at 1 MHz with pulse amplitude modulated by the supply voltage. This demonstrated the capability to miniature TD-NIRS pulsed sources to sizes rivaling CW-NIRS illumination modules. Building on this approach, Jansson *et al* demonstrated the feasibility of integrating a CMOS-based laser driver directly into a compact TD-NIRS optode (figure 8(1)) [169]. The system employed a dedicated illumination PCB featuring a CMOS laser driver and laser diode (6 × 12 mm^2^) that generates ∼80 ps pulses, paired with a receiver IC that integrates a 9 × 9 SPAD array and a 10-channel TDC. The authors demonstrated *in vivo* feasibility by performing a venous occlusion experiment that confirmed sufficient depth sensitivity and timing performance for physiological measurements. While both papers substantially improve the miniaturization of TD-NIRS hardware, the repetition rates of 1 MHz and 500 kHz, respectively, are far below the 50–100 MHz typically seen in state-of-the-art TD-NIRS systems.

Several groups have also pursued fully integrated TD-NIRS transceivers. Saha *et al* presented a fiber-free null SDS optode design integrating pulsed illumination, time-gated detection, and control electronics within a CMOS-based architecture [27]. Iterative designs culminated in a system featuring a VCSEL source (850 nm), a SPAD detector, laser driver, PLL, and time-gating circuitry in a 12 × 12 mm^2^ area housed in a 2 × 1.5 × 1 in^3^ case. More recently, Nissinen *et al* presented a highly integrated single-chip CMOS TD-NIRS transceiver fabricated in a 150 nm process [170]. The 6 × 5 mm chip integrates an 8 × 32 SPAD array with 32 TDCs offering ∼60 ps resolution, alongside a laser diode driver capable of producing ∼0.5 A pulses at 15 MHz. However, since the device was not formally characterized using phantom or *in vivo* based studies, its application in real-world scenarios has yet to be determined. Though with a smaller SPAD array and fewer TDCs, Moazeni *et al* produced a similar highly integrated SoC (1.8 × 1.8 mm^2^ chip size) with a repetition rate of 100 MHz [28]. This level of monolithic integration that combines photon detection, time-resolved readout, and high-current source driving marks a significant advancement toward wearable TD-NIRS, but this comes at the expense of a SPAD array with a low fill factor (3%) resulting in much of the chip area within the array not being light-sensitive, which drastically reduces PDE. Further progress is expected from the next generation of this device which is in development [171].

Overall, recent advances in CMOS integration demonstrate a clear path toward compact, low-profile TD-NIRS hardware. Since the systems and components described here are not yet fully wearable and wireless, continued progress in system-level integration and experimental validation represent the next steps to transition TD-NIRS from laboratory prototypes to low-profile, fully wearable platforms suitable for continuous monitoring.

#### 4.2.2 Compact and wearable TD-NIRS devices

Parallel efforts have focused on enabling time-resolved imaging in naturalistic settings through the creation larger, body-worn platforms. A showcase example is the battery-operated backpack-based system developed by Lacerenza *et al*, shown in figure 8(2a) [30]. At a size and weight of 200 × 160 × 50 mm^3^ and ∼2500 g, the system integrates two laser diodes operating at 670 nm and 830 nm, a SiPM with an active area of 1.3 × 1.3 mm^2^, and a TDC ASIC providing 10 ps temporal resolution. The lasers operate in gain-switching mode to generate short 205 ps optical pulses suitable for TD measurements, while a 14.8 V lithium ion battery allows up to 6 h of continuous operation. The system was extensively validated using solid homogeneous and heterogeneous tissue-simulating phantoms as well as *in vivo* finger tapping, breath holding, and outdoor bike riding paradigms. This implementation highlights the feasibility of TD-NIRS measurements during unconstrained movement and real-world activities, though it is too large for comfortable long-term continuous monitoring.

The continued development of the backpack-based architecture described above has led to the only commercially available TD-NIRS system offering battery-operated functionality: the NIRSBOX (PIONIRS, Italy) [173] shown in figure 8(2b). NIRSBOX is a compact, dual-wavelength, two-channel system marketed to monitor cerebral and muscle oxygenation, functional neuroimaging, and optical evaluation of food quality such as vegetables. Despite the possibility of battery-powered operation, NIRSBOX is still fiber-coupled and requires a USB connection for data transmission. Combined with its overall system bulk, these constraints restrict its practicality for studies involving prolonged naturalistic monitoring.

Although strictly not wireless, since it relies on wired power and tethered data transmission, it is still worth highlighting the commercial Flow2 system (Kernel, USA) shown in figure 8(3) [83]. The Flow2 system represents a substantial engineering feat that has translated HD-TD-DOT into a self-contained, helmet-based form factor. It features 40 modules covering the frontal, parietal, temporal, and occipital cortices. Each module includes three dual-wavelength laser sources (690 and 850 nm) and six detectors arranged around an additional detector, resulting in intra-module SDS of 8.5 mm, 17.9 mm, and 26.5 mm. Light is transmitted and received via spring-loaded light pipes with custom lenses to effectively guide light to and from tissue. The size of the headset is adjustable for adult heads and the system weighs 2400 g, powered by USB-C. Also enabled by custom TD-NIRS ASICs, the system is an impressive demonstration of how to handle practical constraints (e.g. thermal management, power delivery, optode density, weight, ambient light interference, and system complexity) that accompany the packaging of large field-of-view TD-NIRS neuroimaging in a compact format.

### 4.3 FD-NIRS devices

**Figure 9.**
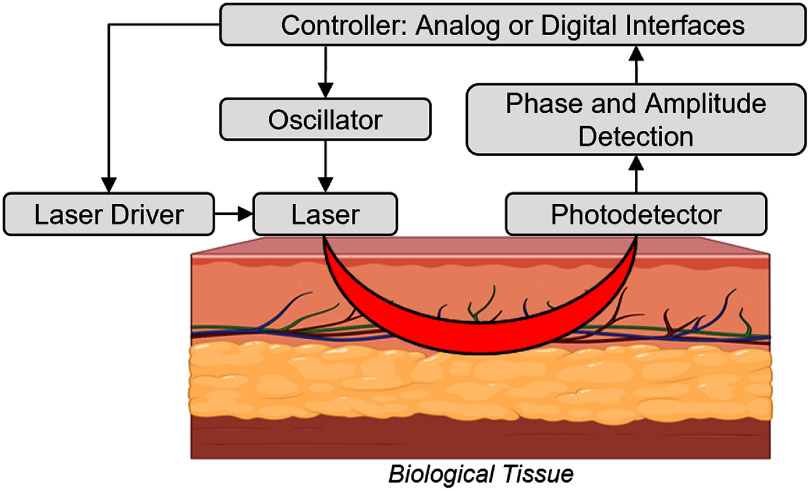
Architecture of an FD-NIRS system. A central controller manages the high-frequency RF oscillator and DC bias (laser driver) signal paths, which are combined to provide a modulated optical signal that is delivered to the tissue. The remitted optical signal is captured and demodulated to extract the attenuation (amplitude) and phase shift—or equivalently, real and imaginary components of the photon density wave. These parameters are digitized for subsequent analysis of tissue optical properties using model-based computational approaches.

A schematic block diagram for an FD-NIRS device, which uses intensity-modulated laser light to measure tissue optical properties, is shown in figure 9. FD-NIRS systems must generate high frequency (single or multiple modulation frequencies in the range of 50–1000 MHz) laser modulation, utilize high bandwidth sensitive photodetectors to detect light after attenuation through tissue, and a demodulator to measure the resulting amplitude and phase. Heterodyne and IQ modulation/demodulation are common approaches for FD-NIRS detection, although others have been demonstrated [15]. Early FD-NIRS systems were bulky and complex, but significant progress has been made in developing compact [174, 175], handheld [42], and wearable tethered [49] FD-NIRS systems.

#### 4.3.1 Advances in FD-NIRS ASIC technologies

To realize fully wearable and wireless FD-NIRS devices, like for TD-NIRS, recent efforts have focused on creating ASICs to manage the FD signal chain. Since FD-NIRS sensitivity tends to be limited by detector performance, the receiver analog front end (AFE) in particular plays a critical role. Monolithic integration of photodetectors with pre-amplifiers (e.g. TIAs) eliminates the need for external wires, reducing parasitic capacitance, improving noise performance, and lowering power consumption [176, 177]. Atef *et al* demonstrated this with PIN photodiodes [177, 178], however the resulting detector bandwidth ($ {\gt} $20 MHz) was somewhat low for FD-NIRS and would require exceptionally high phase precision to accurately measure tissue optical properties. In another example, Yun *et al* integrated APDs with frequency-mixing TIAs in one stage to achieve 100 MHz modulation, while also reducing power consumption [44]. While each work focuses on the detection portion of the FD-NIRS signal chain, neither report phase resolution capabilities which is a defining characteristic for FD systems and impacts absolute optical property estimation.

Accurate and precise phase measurements remain a defining challenge for the design of FD-NIRS ASICs. Because optical signals attenuate exponentially with distance, the receiver must support a wide dynamic range to maintain sufficient SNR across varying SDS [178]. To avoid saturation, pseudo-logarithmic amplifiers (log-amps) have been implemented using arrays of saturation amplifiers with summed outputs to compress the input current into a linear output voltage [73, 179]. Alternative methods have also been reported, including amplification followed by peak-detection using track-and-hold circuits with subsequent digitization via ADC [180] and downconversion followed by filtering, amplification, and ADC-digitization [181]. Since phase detection relies on the output from the AFE receiver, how the amplitude is managed strongly impacts phase resolution, bandwidth, and power efficiency. Consequently, the need to accommodate a wide dynamic range plays a key role in selecting the appropriate phase extraction strategy. Conventional XNOR-based phase detection [182] requires amplitude-limited square waves, which require power-hungry multi-stage limiting amplifiers that can degrade phase accuracy. Consequently, FD-NIRS ASIC development over the past ∼15 years has explored low-power topologies, including down-conversion mixers (e.g. a Gilbert mixer) for IQ demodulation [183], TDC approaches [184, 185], N-path filtering for phase comparison during unequal amplitudes [186], delay-locked loop strategies that uses delay elements to match channel phases [180], and on-chip comparators and flip-flop zero-crossing timing methods [187]. Recently, Ma *et al* demonstrated a 1-bit $\Sigma-\Delta$ phase to digital converter achieving sub-0.01^∘^ resolution, enabling high precision measurements appropriate for cerebral oximetry [188].

**Figure 10.**
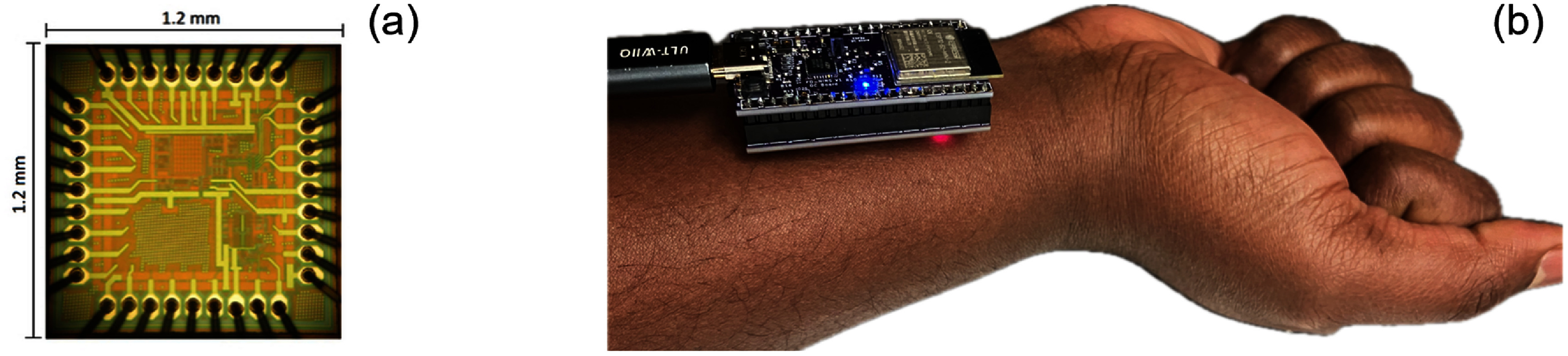
(a) FD-NIRS broad-bandwidth ASIC chip which is integrated into a (b) wearable broad-bandwidth FD-NIRS device by Lahade *et al* [49]. Reprinted with permission from [49] © The Optical Society.

Because the oscillator and RF signal chain define much of the FD-NIRS hardware footprint, integrating RF generation (50–1000 MHz) on-chip is an advantageous step toward miniaturized FD-NIRS systems. Illustrated in figure 10(a), Yazdi *et al* realized an on-chip oscillator, which combined multiple AFE TIA and variable gain amplifier (VGA) stages to achieve a dynamic range of 60 dB [73]. This ASIC was integrated into the wearable FD-NIRS system described in section 4.3.2 and shown in figure 10(b). Another important integration strategy is the incorporation of on-chip laser drivers for improved power management. For example, laser drivers integrated into a 180 nm CMOS ASIC and controlled by a digital core have reported to efficiently drive laser sources for FD-fNIRS operation [180]. Finally, recent FD-NIRS ASICs have been extended beyond sensing alone to enable integrated therapy and closed-loop intervention. A prime example is a 130 nm CMOS system that integrates FD-NIRS with transcranial direct-current stimulation (tDCS) using a programmable voltage-controlled resistor stimulator [183].

#### 4.3.2 Fully wearable multi-frequency FD-NIRS

Lahade *et al* recently demonstrated a compact fiberless wearable FD-NIRS device (67 × 27 × 28 mm^3^, 37 g) that achieves accurate quantitative tissue characterization (figure 10(b)) [49]. The core of this architecture is a custom $0.18\,\mu$m BiCMOS ASIC that performs on-chip multi-frequency synthesis and extracts amplitude and phase shift through an integrated signal chain comprising a TIA, VGA, and linear phase detectors. The SiPM photodetector requires a relatively low bias voltage of 52.6 V, significantly reducing the footprint and power requirements for high-voltage conversion components. The multi-frequency, dual-wavelength (685 and 850 nm) system operates across a 50–350 MHz modulation range and validation using tissue-simulating phantoms yielded high accuracy, including a root mean square error of $ {\lt} 0.001\,\mathrm{mm}^{-1}$ for absorption and $ {\lt} 0.1\,\mathrm{mm}^{-1}$ for reduced scattering coefficients. The system’s performance was validated by tracking absolute hemoglobin changes during a forearm venous occlusion challenge at 14.7 Hz and its high-speed sensing capabilities by capturing pulsatile signals at 250 Hz. The device communicates over Wi-Fi and is currently powered by a USB-C cable, however, this can be replaced with a portable battery for fully wearable wireless operation. This work represents a significant advancement in transitioning quantitative FD-NIRS to self-contained, mobile-controlled wearables suitable for naturalistic environments.

### 4.4 Multimodal integration of wearable NIRS with other health sensing technologies

While ongoing hardware advances continue to improve the performance of standalone wearable NIRS systems, many clinical and research questions benefit from information beyond what NIRS can provide. Consequently, this section provides an overview of the work to integrate NIRS with complementary sensing and stimulation modalities that improve temporal resolution, expand spatial context, investigate additional physiology, and deliver treatment. These multimodal approaches aim to provide a more comprehensive characterization of tissue physiology by combining the strengths of different technologies within unified wearable platforms [189]. Most commercial multimodality approaches combine information gathered from disparate systems (i.e. in software) without close hardware integration. We instead focus our review on instances of low-level integration, specifically approaches that combine fNIRS with EEG and transcranial stimulation, as well as NIRS with electromyography (EMG) and ultrasound (US).

#### 4.4.1 fNIRS + EEG

By placing electrodes in contact with the scalp, EEG noninvasively records the electrical activity of the brain and has been used extensively for seizure detection [190], epilepsy evaluation [191], and diagnosis of sleeping disorders [192]. EEG-fNIRS integration is motivated by the complementary strengths of the two modalities: EEG provides millisecond-scale temporal resolution but limited spatial specificity, while fNIRS offers improved spatial localization at the cost of slower hemodynamic responses. Advances in instrumentation have enabled concurrent acquisition during movement and in naturalistic settings, motivating tighter physical and electronic integration between modalities [193, 194]. Early work emphasized minimizing system footprint by co-locating optodes and electrodes at the scalp interface, exemplified by ear-mounted and head-worn platforms that integrate optical sources, detectors, EEG front ends, and wireless telemetry within compact form factors [195, 196]. Subsequent designs refined this approach through explicit mechanical co-design of electrode-optode interfaces, demonstrating that dense co-location can be achieved without degrading EEG spectral fidelity or optical signal quality [197, 198].

Parallel efforts explored shared acquisition architectures in which EEG and fNIRS channels are synchronized through common timing, ADCs, or modular electronics, enabling scalable multimodal coverage across the head [71, 199, 200]. More recent systems have emphasized increased channel count and sampling rates within single wearable modules while maintaining synchronized acquisition across modalities [36]. Collectively, these approaches illustrate a progression from proof-of-concept co-measurement toward increasingly integrated, wearable EEG–fNIRS platforms, although complexity, noise pickup, MAs, and data interpretation remain barriers to clinical translation.

#### 4.4.2 fNIRS + Transcranial stimulation

The combination of fNIRS with transcranial stimulation techniques, such as tDCS, alternating current (tACS), and magnetic (TMS) stimulation, enables simultaneous modulation and monitoring of cortical activity, supporting studies of cognition, rehabilitation, and neuropsychiatric disorders [201, 202]. By applying low-intensity current (tDCS or tACS) or electromagnetic pulses TMS to the brain via scalp electrodes or an external coil, respectively, neurons can be stimulated or modulated to alter and regulate neural activity [203]. While many studies pair commercial stimulation and fNIRS systems, fewer efforts address hardware-level integration required for wearable or closed-loop operation. Early work demonstrated that fNIRS sensors could be designed to operate under active stimulation by mechanically and electrically accommodating stimulation electrodes without compromising optical signal quality [204]. Flexible probes utilizing silicone-encapsulated designs have allowed for close spatial integration of optodes and electrodes while mitigating stimulation-induced artifacts.

More advanced efforts moved beyond compatibility toward true co-integration. Custom ASIC front ends have been developed that unify EEG amplification, dual-wavelength NIRS readout, and reconfigurable stimulation current sources within a single chip, providing concurrent sensing and stimulation within compact head-worn platforms [195, 196]. These architectures represent a shift from modular multimodal systems toward unified hardware stacks capable of real-time monitoring during neuromodulation. Together, these efforts chart a trajectory toward wearable, closed-loop neurostimulation platforms in which sensing and stimulation are coordinated at the hardware level.

#### 4.4.3 NIRS + EMG

NIRS-EMG integration extends multimodal sensing beyond the brain to skeletal muscle, combining electrophysiological measurements of motor unit activation with optical assessment of muscle oxygenation. Since early demonstrations in the mid-1990s [205], these dual modalities have been applied for applications including rehabilitation [206, 207], endurance monitoring [208, 209], and prosthetic control [210, 211]. While many studies rely on synchronized but independent devices, a smaller body of work has pursued hardware-level integration to improve timing control and wearability. Early integrated probes contained optical and electrical sensors co-packaged into modular units. For example, when these units were distributed around a limb, their sensing of real-time NIRS and EMG features resulted in improved performance in a prosthetic control application [212, 213].

Recent designs have further pushed integration through flexible, layered sensor architectures that embed optical paths and surface electrodes within a single thin probe, enabling wireless operation and dense multimodal sampling [214]. Extensions to tri-modal sensing, incorporating mechanomyography alongside NIRS and EMG within a unified probe, highlight the potential for rich physiological monitoring without significantly increasing hardware complexity [215]. Despite these advances, most NIRS–EMG systems remain tethered or semi-wearable, underscoring ongoing challenges in power management, wireless communication, and long-term deployment.

#### 4.4.4 NIRS + US

US provides structural tissue information that complements the functional contrast offered by NIRS for a more complete picture of tissue physiology. Adding US can be beneficial for applications that require anatomical localization to guide the positioning of the NIRS probe and can be used to constrain NIRS-based imaging in DOT [216]. Previous work has used US guidance to inform optical measurements in placental and breast imaging, as well as to monitor cortical hemodynamic responses after transcranial focused US neuromodulation [217–219]. In these contexts, NIRS serves as a noninvasive readout of vascular or metabolic changes induced or localized by acoustic interrogation. However, it should be considered that functional optical contrast does not necessarily exactly co-register with structural US contrast.

Translation of NIRS-US systems into wearable or fully wireless platforms remains limited. Most reported implementations rely on handheld probes tethered to bench-top US instrumentation, restricting mobility and long-term use [216]. We identified only a single wireless NIRS–tFUS system, which required surgical fixation and therefore falls outside the scope of noninvasive wearable technologies [220]. With the advent of new wearable US systems [221], NIRS–US integration remains an emerging area with substantial potential for innovation and new functionality.

### 4.5 Wearable device summary

While the wearable hardware architectures and demonstrations described in this section represent significant engineering milestones, their real-world utility is often governed by practical constraints such as wearability, MAs, and signal interference. We provide our evaluation of these challenges and proposed mitigation strategies in section 6.1, following a review of the primary clinical applications enabled by these devices.

## 5 Human applications of wearable NIRS devices

NIRS has been widely applied as tools in neuroscience research, clinical monitoring, and sports science. For example, by tracking changes in cerebral hemodynamics and tissue blood oxygen saturation, researchers and clinicians use NIRS to map and evaluate functional brain activity [222–225], identify and monitor cerebral ischemia [226, 227], analyze muscle composition and metabolism [228], and even examine breast cancer progression and therapy response [229–231]. In this section, we discuss the use of wearable NIRS in various human applications, emphasizing how experimental demands inform hardware architecture and system-level tradeoffs.

### 5.1 Functional imaging and sensing of the brain

#### 5.1.1 fNIRS in naturalistic environments

Functional brain imaging is one of the main applications driving the development of wearable NIRS and fNIRS systems. By measuring task- or stimulus-evoked changes in cerebral hemodynamics arising from neurovascular coupling, wearable fNIRS enables the study of cortical activity during everyday life, continuously over long periods, and in environments that are not suited to conventional neuroimaging modalities such as fMRI. As a result, functional brain imaging has placed some of the most stringent hardware, ergonomics, and signal-quality requirements on wearable NIRS device design.

One of the primary appeals of fNIRS lies in its potential for neuroimaging in unconstrained settings such as the home, workplace, or during athletic activity. While laboratory-based studies provide immense insight into brain function, they do not fully capture how cortical networks are engaged during everyday life. Wearable fNIRS has been used to investigate changes in functional connectivity during physical activity across different environments (e.g. outdoor versus indoor, moving versus stationary) [37], to identify predominantly active networks during unpredictable athletic play [232], and to study brain activation during fine or complex motor tasks such as playing piano or violin [232].

From a hardware perspective, complete untethered operation, and therefore the inclusion of onboard storage or wireless data transmission, is pertinent for free range settings. In outdoor paradigms, ambient light blocking measures are a critical requirement preserving signal quality. Self-contained systems integrated into a cap or headband reduce motion-induced artifacts associated with cabling but introduce tradeoffs between spatial coverage, channel density, and device weight. While wide field-of-view configurations are desirable for investigating functional connectivity, increased channel count directly impacts mass and comfort. Considerations related to widespread adoption also include ease of self-placement and minimal reliance on trained operators and data specialists. For home use, forehead-mounted probes offer a practical compromise by reducing hair-related coupling issues, and recent efforts have explored app-based guidance for self-administered placement, signal quality checks, and task execution [133].

#### 5.1.2 Brain computer interface (BCI)

Wearable fNIRS has been explored as an interface for assistive control and neurofeedback. fNIRS-based BCIs [52] have been investigated for communications for locked-in syndrome [233, 234], motor intention for assistive devices [211, 235, 236], and executive functions for passive BCI paradigms. Additionally, fNIRS has been utilized in closed-loop systems incorporating neurofeedback aimed at modulating and optimizing neural activity during learning and performance-based tasks [237–239].

These applications typically require real-time data acquisition and processing, putting additional constraints on system latency, onboard computation, and wireless bandwidth. Hardware platforms used in this space frequently prioritize low-latency signal processing over dense spatial coverage. To reduce latency and improve temporal resolution, hybrid EEG-fNIRS BCI architectures have been explored and have shown to improve classification performance when compared to EEG or fNIRS alone [240, 241]. Such systems introduce additional complexity related to synchronization and data fusion; however, this can be mitigated with the use of Lab Streaming Layer, a low-latency data fusion software platform [242]. Furthermore, ML has been a focus for enhancing classification accuracy, motivating architectures that incorporate onboard processing units capable of performing computationally intensive tasks and managing large bandwidth [235, 243].

#### 5.1.3 Neurorehabilitation

Neurorehabilitation aims to restore motor, cognitive, and sensory function following stroke, traumatic brain injury, or neurological disease through intensive, task-specific therapy that promotes neural reorganization. Understanding how the brain reorganizes during recovery, and whether therapeutic interventions successfully drive beneficial plasticity, requires longitudinal monitoring of cortical activity during and after rehabilitation exercises, a capability well suited to wearable fNIRS. Neurorehabilitation applications place particular demands on wearable fNIRS systems, including robustness to motion, repeatable optode placement across sessions, and long-term stability for longitudinal monitoring. These requirements are particularly evident in post-stroke rehabilitation studies, where fNIRS has been used to monitor activation and track neuroplasticity during motor recovery and therapy sessions [207, 244, 245]. Several wearable systems, such as Artinis Brite and NIRx NIRsport, have been deployed in this context to monitor task-evoked hemodynamic responses during upper- [246, 247] and lower-limb rehabilitation [248, 249]. Wearable fNIRS has also been used to evaluate the effectiveness of neurostimulation therapy such as tDCS [203, 250], TMS [203], and neuromuscular electrical stimulation [251]. fNIRS can be used to optimize activation in regions of interest, leading to improved rehabilitation outcomes.

#### 5.1.4 Mental health and psychiatric monitoring

Wearable fNIRS has been explored in studies of depression [252], anxiety, schizophrenia [253, 254] and attention-deficit/hyperactivity disorder [239]. Prior work suggests that fNIRS signals may provide predictive information regarding treatment response, although many studies utilize tethered or laboratory-based devices [255–257]. From a hardware perspective, these studies emphasize the value of lightweight, low-profile, and hardened designs that support prolonged and repeated use, as studies can last six months to a year [255]. Forehead-mounted designs are common due to the focus on prefrontal regions, however, findings implicating occipital and parietal cortices in depression motivate devices capable of reliable coupling through hair [255, 258]. Aesthetic considerations may also influence user compliance, particularly for long-term monitoring in sensitive populations.

#### 5.1.5 Neurodegenerative diseases

In studies of aging as well as neurodegenerative disease, wearable fNIRS has been used to assess task-evoked and resting-state cerebral hemodynamics as potential markers of cognitive impairment such as Alzheimer’s/dementia disease (AD) [259–261] and movement disorders such as Parkinson’s disease (PD) [262–265]. These studies often involve older adults with increased sensitivity to probe pressure, longer preparation times, and greater inter-session variability, placing unique constraints on device ergonomics and usability.

Applications in this domain highlight the importance of ergonomic optode designs, low-force optical coupling, and simplified cap architectures that can be deployed reliably across repeated clinical visits. While reduced-channel-count systems are commonly adopted to minimize preparation burden and improve comfort, wide field-of-view configurations may be advantageous for detecting distributed cortical changes associated with cognitive decline and AD [261]. Greater standardization in hardware and protocols will be essential for greater clinical adoption and to enable studies of disease prognosis and treatment response [266].

#### 5.1.6 Cerebral oximetry

In contrast to fNIRS systems designed for cortical activity mapping, cerebral oximetry typically employs flexible sensors to provide continuous measurement of regional cerebral oxygenation (rScO_2_). They have been used to detect hypoxic events in surgical (e.g. cardiac) and neurocritical settings, allowing quick interventions aimed at reversing desaturation and improving patient recovery times and outcomes [104, 154, 267]. Furthermore, cerebral oximeters are one of the few classes of NIRS instruments that have gained marketing clearance from the Food and Drug Administration in the USA [268, 269], reflecting both their clinical utility and the maturity of the technology. However, these devices require cabling that adds to the preexisting cables, cords, and tubes from other critical care machinery, a phenomenon commonly referred to as ‘spaghetti syndrome’. The proliferation of wired connections can lead to falls by both personnel and patients, damage to medical equipment, and in some cases death of patients [270–272].

Addressing spaghetti syndrome motivates the development of wearable NIRS devices with integrated wireless communication modules capable of providing stable data transmission with minimal latency for hours at a time [104, 132, 154, 273]. For prolonged monitoring applications, low-power components and low-duty cycle modes are essential to extend battery life and reduce device temperature [77, 104, 154, 273–275]. Battery management strategies that allow rapid battery exchange can further minimize interruptions in monitoring [104].

### 5.2 Exercise and sports medicine

Wearable NIRS hardware has found new applications in sports science. In these settings, NIRS devices have been used to monitor muscle oxygenation during exercise, evaluate the influence of exercise on cognitive function, and inform training and rehabilitation strategies aimed at improving productivity while minimizing injury risk. By noninvasively tracking changes in myoglobin and hemoglobin concentrations within superficial muscle, wearable NIRS muscle oximeters enable continuous measurement of local SmO_2_ during dynamic activities such as walking, resistance exercise, cycling, and therapeutic movement [163]. These measurements have proven valuable for evaluating functional capacity and recovery in older adults undergoing rehabilitation [276, 277], as well as for quantifying physiological responses to training regimens, muscle stimulation, and fatigue in athletic populations [64, 164, 278–280].

Driven by the demand for field-deployable monitoring tools, wearable muscle oximeters have been developed with an emphasis on compact form factor, wireless data transmission, and robustness during high motion tasks [64]. Commercially available systems have demonstrated the ability to capture trends during exercise and occlusion paradigms [163, 164], supporting their use as practical tools to monitor changes in muscle oxygenation. However, validation studies have also highlighted limitations in cross-session repeatability and sensitivity to subject-specific factors such as subcutaneous adipose thickness and tissue heterogeneity, reinforcing the challenges associated with translating NIRS muscle oximetry across diverse populations and conditions [163, 280, 281]. These limitations motivate continued attention to hardware design and algorithmic strategies that can improve measurement robustness and reliability across all users.

Wearable muscle oximetry presents a unique set of challenges compared to cerebral applications. Skeletal muscle undergoes repeated compression, expansion, and geometric deformation during contraction and relaxation cycles, requiring devices to maintain stable optode–tissue coupling under continuously changing mechanical conditions [163]. Consequently, flexible substrates and mechanically robust optode mounting strategies–including flexible patches, compression sleeves, and even ‘smart textile’ approaches–are critical to preserving signal quality during prolonged or high-intensity activity [64, 279, 280, 282, 283]. Beyond mechanical robustness, mounting techniques must prioritize user comfort, as user satisfaction is essential for widespread adoption. Successful architectures must improve user experience by providing information in an easily interpretable format with actionable insights [280].

SDS’s and optical power levels must also be carefully selected to balance sensitivity to underlying muscle tissue while mitigating contamination from overlying skin and adipose layers, particularly in clinical and non-athletic populations [163, 280, 281]. Tissue thickness information from complementary imaging, such as US, can be used in a clinical setting to improve muscle sensitivity, however, this may not available to a non-clinical user unless it is fully integrated with the NIRS device. Since muscle oximetry protocols often involve extended monitoring periods during training sessions or rehabilitation exercises, additional considerations include power efficiency and data reliability. Low power electronics, efficient wireless communications, and thermal management strategies remain essential to ensure uninterrupted operation over prolonged use [64].

As wearable muscle oximetry continues to mature, standardized calibration approaches and consistent data interpretation frameworks will be necessary to improve reproducibility between devices, sessions, and subject groups, ultimately strengthening its utility in both performance optimization and clinical decision making [281]. For a comprehensive overview of muscle oximetry applications, validation efforts, and commercially available systems, the reader is directed to the recent review by Perrey *et al* [163].

### 5.3 Other human applications

Beyond the dominant applications in neuroscience and muscle oximetry, wearable NIRS has been explored for diverse clinical and diagnostic contexts that leverage its ability to noninvasively assess tissue oxygenation and hemodynamics. In wound care, NIRS-based assessment of tissue oxygenation provides prognostic information for diabetic foot ulcers, where direct perfusion impacts healing outcomes [284]. Urological applications exploit NIR light transmission through the abdominal wall to provide noninvasive bladder volume monitoring, potentially offering an alternative to catheter procedures for individuals with neurogenic bladder or urinary retention [285]. Similarly, noninvasive glucose monitoring leverages NIR spectroscopy’s sensitivity to blood constituents, though challenges related to measurement specificity and calibration stability remain active areas of investigation [286]. Breast imaging applications leverage NIRS-based quantification of tumor hemoglobin concentration for early cancer detection and chemotherapy response monitoring [55, 287, 288].

## 6 Perspective and needs for the future of wearable NIRS

As wearable NIRS technology continues to mature and expand into existing and new application domains, several technical and translational challenges require sustained attention. In this section, we summarize what we believe are the major challenges and opportunities for wireless wearable NIRS and identify research directions to address them. Together, we hope that this will help drive the widespread adoption of wearable NIRS for both clinical and consumer applications that improve the human condition.

### 6.1 Device design improvements

#### 6.1.1 Design for comfort, reliability, and long-term use

Our vision for the future of wearable NIRS includes unobtrusive, yet highly capable devices that disappear into daily life: advanced quantitative NIRS methods that are highly integrated with consumer wearables (smart watches, rings, etc), thin skin-conformal NIRS devices that are tattoo-like, and NIRS-integrated garments that users forget they are wearing. The recent success of the Oura Ring finger-worn wearable, which utilizes PPG optical sensing for health monitoring, already demonstrates the feasibility of integrating and mass producing wireless battery-powered devices with ultra-small integration of optical sources and detectors [289].

Achieving this requires continued advances in miniaturized components, as well as flexible substrates such as ultra-thin polyimide films [290–292] and medical-grade silicones [104, 291] with stretchable interconnects [104, 291] that accommodate tissue deformation during movement while maintaining robust functionality. Smart textiles incorporating conductive yarns and washable electronics [163, 283] could transform everyday clothing into continuous monitoring platforms, although challenges remain in optode positioning repeatability and optical coupling stability during fabric stretch [283, 293]. The ultimate goal should be to create long-lasting effective wearable NIRS systems that are lightweight, comfortable, and easy-to-operate for continuous monitoring over days to weeks.

We expect that the next generation of miniaturized components for NIRS will benefit from the high-volume manufacturing of heterogeneously integrated optoelectronics (e.g. lasers, LEDs, and photodetectors) with silicon electronics at the chip level. This capability can produce dramatic reductions in footprint and weight while improving reliability through reduced interconnect complexity. At the same time, applications for wearable NIRS can be expanded through the development of accessible miniature light sources that operate at extended wavelengths (e.g. SWIR and longer) or can be wavelength-tuned. Deep tissue sensing can be improved through the use of low-bias, low-noise, and sensitive photodetectors, while integrated optical filters can lower susceptibility to ambient light interference–especially important when these devices are designed for outdoor use.

Effective tissue optical coupling is critical for robust wearable NIRS operation, especially in the presence of movement. At the optode level, micro-optic integration such as gradient-index lenses for compact focusing, microlenses for improved light collection, or diffractive optical elements for beam shaping [83] could enhance SNR while reducing required optical power. The incorporation of photonic integrated circuits could lead to the development of fully integrated NIRS systems-on-chips that consolidate sources, detectors, optics, and control electronics into millimeter-scale packages. These ‘NIRS-on-a-chip’ components could be used individually or in tandem to create multi-channel and HD NIRS sensing arrays.

Realizing truly untethered, long-duration monitoring requires re-imagining power and thermal architectures. Ultra-low-power ASICs operating in sub-threshold regimes [294] with duty-cycling [77, 274, 275] and dynamic power scaling could extend battery life. Advanced solid-state batteries hope to offer improved energy density and safety [295–297] while hybrid supercapacitor architectures for peak power delivery [298] could support pulsed illumination schemes. Incorporating low power standby and sleep modes is critical to mitigate the high power draw from optical sources. Energy could be harvested from body heat via thermoelectric generators, motion through piezoelectric elements, or sunlight via flexible photovoltaics [299] to supplement or eliminate battery dependence.

However, reducing power consumption alone is insufficient; heat generated by optical sources, drive electronics, and batteries must also be managed carefully, as thermal considerations affect both user safety and device performance. Good thermal management is often at odds with flexible materials that tend to be insulating, creating trade-offs between flexibility and functionality. At the tissue-device interface, the International Electrotechnical Commission (IEC) 60601-1 standard specifies that the temperature of device surfaces contacting the skin for over 10 min should be less than 43 ^∘^C for safe operation [300]. Innovative solutions to address thermal management include the use of phase-change and high thermal conductivity materials to dissipate heat [301], while strategically placing power-hungry components could avoid the need for bulky heatsinks or thermoelectric cooling. Insulating barriers such as thin acrylic or silicone layers can provide safeguards against localized hotspots. Thermal management is not only important for safety, but also stable and reliable operation. For example, laser center wavelengths can drift 0.2–0.3 nm ^∘^C^−1^ [302], and the dark current of photodetectors are temperature dependent.

#### 6.1.2 Addressing hair and skin tone

Correcting for the effects of skin and hair tone on NIRS measurements remains an active area of research. The equitable widespread deployment of wearable NIRS technology requires devices to handle melanin-related wavelength-dependent signal attenuation and hair interference issues, which are problems that have plagued accurate measurements in some individuals [163, 303, 304]. The degree by which skin tone affects NIRS depends highly upon the specific NIRS device design utilized (e.g. CW vs time-resolved, estimating absolute vs relative changes, optode layout, single vs multi-distance techniques). Some time-resolved implementations (TD and multi-distance FD) have demonstrated reduced melanin sensitivity [20, 305]. Multi-wavelength strategies to account for the absorption profile of melanin is a promising approach [304, 305], but it must be balanced against increased complexity and reduced sampling rates. In any scenario, it is essential to evaluate NIRS device performance using appropriate tissue phantoms that mimic the full range of skin tones [20]. To assure optical coupling through diverse hair types, brush optodes are advantageous [88] but remain under-validated, while custom hair-clearance cap attachments show promise [85]. In clinical studies, it is essential to record skin tone using objective quantitative measures (i.e. melanometry [306]), and consider device performance within its context. Similarly, hair attributes (e.g. color, thickness, type) must be considered if applicable.

#### 6.1.3 Mitigating MAs

Despite recent progress in MA correction methods and hardware-assisted mitigation strategies discussed in section 3.4, achieving robust performance during unrestricted daily activities remains an unsolved challenge. The field needs standardized validation frameworks and benchmark datasets that enable rigorous comparison of devices and correction strategies. Currently, performance claims vary widely between studies and they utilize inconsistent evaluation criteria. More fundamentally, realizing real-time motion correction that generalizes across different movement patterns while preserving NIRS signals would benefit from new approaches. For example, adaptive ML algorithms that continuously learn subject-specific artifact signatures, potentially combined with advanced hardware stabilization, could close the gap between controlled laboratory performance and naturalistic deployment. Motion-tolerant systems that enable continuous brain and muscle monitoring during unrestricted activities–walking, exercising, sports–without sacrificing signal quality would unlock the full potential of wearable NIRS for ecological validity in neuroscience research, real-world clinical monitoring, and athletic applications.

#### 6.1.4 Scaling to high density configurations

HD NIRS arrays that enable wider tissue coverage with higher spatial resolution face physical and computational scaling challenges as channel counts increase from tens to hundreds of optode pairs. Modular tile-based architectures [101, 106, 143, 307, 308] offer scalability but introduce challenges in synchronization and bandwidth management. Hybrid multiplexing strategies that combine time, frequency, and spatial-division schemes alongside less-explored code-division approaches [309] could manage complexity while maintaining sampling rates, although computational overhead requires attention. As mentioned in section 3.1.4, ASIC integration can consolidate control logic, acquisition, processing, and communication onto single chips dramatically reduces component count and weight for higher density integration. Balancing spatial density against wearability, power budgets, and data throughput requires careful co-design of optical layouts, electronics, and mechanical packaging.

### 6.2 Novel application domains

We have discussed how neuroscience research is currently the primary driver of demand for wearable NIRS devices, while muscle sensing appears to be growing in use and accessibility. Beyond this, we believe that several emerging applications present compelling opportunities for wearable NIRS deployment.

Promising applications in maternal and fetal health include continuous, non-invasive assessment of placental oxygenation [159, 218], fetal hemodynamics [310], maternal-fetal monitoring during labor and delivery, and postpartum hemorrhage. These are significant opportunities given that ∼260,000 women around the world die annually due to pregnancy-related complications, with an estimated 92% of these deaths occurring in low- to lower-middle income countries where access to sufficient maternal care is limited [311]. In the United States, there are still more than 2 million women of child bearing age living in maternity care ‘deserts’ [312]. In addition, recent work showing that NIRS methods are sensitive to lactation physiology is promising for assessing breastfeeding difficulties [313]. Wearable NIRS could thus offer an accessible monitoring tool for improving pregnancy and postpartum care.

Wearable oximetry could provide early detection of cognitive decline and impairment [259, 260] or vascular dementia [266] through longitudinal tracking. This is especially valuable for aging populations living in rural areas that have a limited number of hospitals and even fewer providers specialized in neurology and gerontology. Even in areas with ample healthcare facilities, lack of public transport or other modes of transportation can prevent individuals from getting care [314]. At-home monitoring via NIRS paired with telehealth visits could provide an alternative path to routine neurological care in rural communities.

Urological applications include non-invasive bladder volume monitoring [285], which could improve quality of life for individuals with neurogenic bladder or urinary retention. Neurogenic bladder, a disorder of the lower urinary tract caused by nervous system damage, affects over 40% of individuals with multiple sclerosis, over 70% with spinal cord injuries, and 15% of stroke survivors. It can cause severe complications such as recurrent urinary tract infections and kidney damage [315]. Current management relies heavily on intermittent catheterization, which is invasive, carries a high risk of infection, and requires clinical assistance. For the millions living with neurogenic bladder, wearable NIRS-based bladder monitoring could provide a noninvasive, at-home alternative that reduces catheter dependence and improves independence without requiring frequent clinic visits.

Precise metabolic monitoring is sought after in many sports and rehabilitation settings. The needs include wearable muscle oximetry, lactate threshold tracking, and measurements of oxygen extraction kinetics and recovery dynamics [163]. This is important since ∼8.6 million injuries occur each year due to sports and recreation, with overexertion accounting for ∼1.46 million. Optimizing training intensity, monitoring recovery, and preventing overuse injuries requires more than just personal fatigue assessment. Wearable NIRS could enable real-time metabolic and physiological monitoring during training and rehabilitation, providing athletes with objective data to optimize training regimes, reduce overexertion, and create personalized recovery strategies.

Cardiovascular applications leveraging peripheral NIRS for continuous assessment of tissue perfusion, vascular reactivity [316], or early detection of ischemic events remain underexplored. Critical care monitoring, particularly for hypovolemic or hemorrhagic shock, represents an emerging application space for wearable NIRS technologies. By enabling noninvasive, multi-site tracking of tissue oxygenation and hemoglobin dynamics, wearable NIRS systems may offer complementary physiological insight into regional perfusion and compensatory responses to blood loss, with potential to inform earlier clinical assessment and support resuscitation decision-making in critical environments [317].

### 6.3 Moving to clinical-grade diagnostics

Translating wearable NIRS from a research tool to a clinical diagnostic platform requires surmounting several remaining barriers spanning technical performance, regulatory requirements, standardization, and buy-in from clinicians and healthcare payers. Currently, the lack of universal reference ranges and diagnostic thresholds is driven by significant inter-subject [318, 319] and inter-session [320] variability. These inconsistencies–arising from anatomical differences, tissue heterogeneity, and nonstandardized optode placement–complicate clinical interpretation and thus physician confidence in the tools. Furthermore, the market dominance of CW systems, which are fundamentally limited to relative rather than absolute measurements, further constrains diagnostic utility compared to TD and FD approaches capable of absolute quantification. These technical challenges are compounded by a lack of standardized validation protocols, open-access benchmark datasets, and traceable optical phantoms, all of which impede cross-device comparison and regulatory approval pathways. Ultimately the field requires the identification of specific and validated optical biomarkers that move beyond nonspecific hemodynamic measures to reliably differentiate pathology across diverse populations.

Even technically sound engineering and science cannot advance clinical adoption if the underlying data ecosystem remains fragmented across incompatible proprietary formats and channel labeling conventions. The shared near infrared format (SNIRF) has been endorsed by the fNIRS community and covers CW, FD, and TD implementations [321]. Furthermore, the recently established NIRS Brain Imaging Data Structure (NIRS-BIDS) extension provides the organizational and metadata layer essential for multicenter studies and data sharing [322]. Community-endorsed reporting guidelines also support reproducibility by promoting consistency in documenting acquisition parameters and analysis pipelines [323]. While a detailed treatment of data standards is beyond the scope of this hardware-focused review, developers must recognize that compatibility with SNIRF and NIRS-BIDS may become a prerequisite for clinical and regulatory credibility.

Of course any medical device providing a diagnosis or guiding treatment must adhere to strict regulatory requirements to assure safe operation. This is a necessary step to move from research and consumer grade devices to clinical grade devices. While regulatory pathways for medical devices vary by jurisdiction, they generally require data that the device is (1) safe to use and (2) effective for its intended purpose. For example, IEC 80601-2-71 establishes safety and performance requirements for fNIRS medical devices [324, 325], though its current scope covers only CW systems. Beyond regulatory clearance, securing reimbursement approval from healthcare payers represents a distinct and often formidable barrier. This requires clear evidence of clinical benefit and cost-effectiveness, which have yet to be established for wearable NIRS applications. In our view, this is primarily a consequence of the technical and reproducibility challenges mentioned above, which must be resolved to provide the robust evidence base that payers demand.

Moving forward, multi-modality approaches can be a viable way to address some of the current limitations. Combining the quantitative NIRS techniques–TD and FD–with CW can address some of the reproducibility barriers. Integrating NIRS with complementary modalities such as EEG or EMG can enhance diagnostic specificity by decoupling hemodynamic responses from underlying neural or muscular activity. Incorporating independent measurements of systemic physiological activity, such as heart rate and blood pressure, could also improve the understanding and accurate analysis of NIRS/fNIRS signals [107]. These integrations do not merely add complementary data; they directly address the biomarker specificity problem that currently limits wearable NIRS as a standalone diagnostic tool.

## 7 Limitations

While this review provides an expansive overview of the current progress in the field of wearable untethered NIRS devices and its underlying technologies, we recognize its methodological limitations. Unlike a systematic review, which typically addresses a singular, narrowly defined question [326], this work is structured as a topical state-of-the-art review. This format was chosen specifically to allow for a multi-disciplinary synthesis of hardware design, component-level engineering, device performance, promising clinical applications, and future-looking perspectives that a systematic protocol risks fragmenting. The primary limitation of this approach is the potential for selection bias, as the literature search was guided by our expertise in identifying key engineering and clinical milestones that capture the direction of the field. As such, our narrative approach may not include every study or incremental hardware iteration.

Next, the main goal of this manuscript is to present the hardware innovations that have moved wearable NIRS instrumentation forward and as a result we rarely discuss the computational work that has assisted in this progress. We attempt to acknowledge these advancements in the discussion surrounding mitigating MAs, but identify that a more thorough discussion is needed to fully recognize the algorithmic and computational contributions that have aided in the advancing wearable NIRS to its current state.

Finally, while we cover cerebral, muscle, and several emerging applications, the depth of discussion across these domains is not uniform. Neuroscience and exercise physiology applications benefit from a larger body of wearable NIRS literature and as a result, receive more coverage than areas such as neonatal monitoring, urological, and peripheral vascular assessment. This reflects the current state of the field rather than the authors’ assessment on the importance of these applications.

## 8 Conclusion

Wearable NIRS has progressed rapidly over the past several decades, driven by the promise of continuous, noninvasive monitoring in diverse environments. Advances across CW-, TD-, and FD-NIRS have demonstrated that miniaturized, wireless systems can achieve meaningful sensitivity to cerebral and peripheral hemodynamics under increasingly naturalistic conditions. Yet hardware limitations continue to define the practical limits of current performance in wearable NIRS devices. Tradeoffs between optical power, signal fidelity, motion robustness, channel density, and power consumption remain central constraints, particularly as systems scale toward longitudinal, high density, and clinically relevant deployments.

The most consequential gains in wearable NIRS performance and use will arise from deeper hardware integration rather than incremental system-level refinement. Integrated photonics merging light sources and detectors with silicon electronics, combined with acquisition, control, and processing circuitry via CMOS ASICs, can dramatically reduce the NIRS form factor while improving performance, stability, and scalability. These advances could be particularly impactful for advancing TD- and FD-NIRS, where system complexity has historically limited wearability despite clear advantages in quantitative accuracy. With this, we believe that wearable NIRS is poised to expand beyond current research and limited clinical applications to broader employment. We anticipate that advancements in NIRS technologies will enable new capabilities in high-quality health monitoring across diverse settings and move us closer to the vision of continuous health monitoring integrated unobtrusively into daily life.

## Data Availability

No new data were created or analysed in this study. Supplementary material available at https://doi.org/10.1088/2515-7647/ae6ae4/data1.
